# Candidate gene analysis of spontaneous preterm delivery: New insights from re-analysis of a case-control study using case-parent triads and control-mother dyads

**DOI:** 10.1186/1471-2350-12-174

**Published:** 2011-12-30

**Authors:** Solveig Myking, Ronny Myhre, Håkon K Gjessing, Nils-Halvdan Morken, Verena Sengpiel, Scott M Williams, Kelli K Ryckman, Per Magnus, Bo Jacobsson

**Affiliations:** 1 Department of Genes and Environment, Division of Epidemiology, Norwegian Institute of Public Health, Oslo, Norway; 2 Department of Public Health and Primary Health Care, University of Bergen, Bergen, Norway; 3 Department of Obstetrics and Gynecology, Haukeland University Hospital, Bergen, Norway; 4 Department of Obstetrics and Gynecology, Institute of Clinical Science, Sahlgrenska University Hospital, Göteborg, Sweden; 5 Center for Human Genetics Research, Vanderbilt University, Nashville, Tennessee, USA; 6 Department of Pediatrics, University of Iowa, Iowa City, IA, USA

**Keywords:** case-parent triad analysis, hybrid design, haplotype, pathway analysis, COL5A2, COL5A1

## Abstract

**Background:**

Spontaneous preterm delivery (PTD) has a multifactorial etiology with evidence of a genetic contribution to its pathogenesis. A number of candidate gene case-control studies have been performed on spontaneous PTD, but the results have been inconsistent, and do not fully assess the role of how two genotypes can impact outcome. To elucidate this latter point we re-analyzed data from a previously published case-control candidate gene study, using a case-parent triad design and a hybrid design combining case-parent triads and control-mother dyads. These methods offer a robust approach to genetic association studies for PTD compared to traditional case-control designs.

**Methods:**

The study participants were obtained from the Norwegian Mother and Child Cohort Study (MoBa). A total of 196 case triads and 211 control dyads were selected for the analysis. A case-parent triad design as well as a hybrid design was used to analyze 1,326 SNPs from 159 candidate genes. We compared our results to those from a previous case-control study on the same samples. Haplotypes were analyzed using a sliding window of three SNPs and a pathway analysis was performed to gain biological insight into the pathophysiology of preterm delivery.

**Results:**

The most consistent significant fetal gene across all analyses was COL5A2. The functionally similar COL5A1 was significant when combining fetal and maternal genotypes. PON1 was significant with analytical approaches for single locus association of fetal genes alone, but was possibly confounded by maternal effects. Focal adhesion (hsa04510), Cell Communication (hsa01430) and ECM receptor interaction (hsa04512) were the most constant significant pathways.

**Conclusion:**

This study suggests a fetal association of COL5A2 and a combined fetal-maternal association of COL5A1 with spontaneous PTD. In addition, the pathway analysis implied interactions of genes affecting cell communication and extracellular matrix.

## Background

Preterm delivery (PTD) is defined as delivery occurring before 37 weeks of gestation [1]. In Scandinavian countries PTD rates vary from 5.8% to 6.4% [2]. Children born preterm are at increased risk of neonatal and infant mortality and morbidity. Globally, 28% of neonatal deaths are estimated to be directly attributable to PTD [3]. PTD can be divided into two main groups according to clinical presentation: those with spontaneous onset with either preterm labor (PTL) or preterm prelabor rupture of membranes (pPROM) and those who are delivered due to maternal or fetal complications (e.g. preeclampsia, small for gestational age) [4].

Spontaneous PTD is a common complex condition with no single environmental or genetic factor being completely responsible for its pathogenesis. Known risk factors include infection, inflammation, previous PTD, cigarette smoking, gestational bleeding and low socioeconomic status [5]. Four different pathophysiological pathways have been proposed leading to spontaneous PTD through a common terminal pathway resulting in release of uterotonins and proteases that causes cervical ripening, uterus contractions and membrane rupture [6]. These four pathways are: 1) activation of maternal or fetal hypothalamic pituitary-adrenal (HPA) axis, 2) local or systemic inflammation and infection, 3) decidual hemorrhage and 4) pathological distention of the uterus [6]. Immunological factors, such as abnormal allograft reaction and allergy, have also been hypothesized as possible mechanisms for spontaneous PTD [7]. How each of these putative causal pathways function has been difficult to elucidate.

Epidemiological evidence indicates that genetic factors play a significant role in the etiology of spontaneous PTD [8-11]. A number of candidate gene studies, almost exclusively using case-control design, have identified some genes that associate with PTD [12-17]. However, the results have rarely been replicated. Of importance for this phenotype are the possible effects of two genomes, maternal and fetal, and previous studies have implicated one or the other although epidemiological data supports the predominance of the maternal genome. In addition, interactions between maternal and fetal genomes may affect PTD risk. There has also been uncertainty about the role of the paternal genome [8,11,18-20].

In the present study we re-analyzed data from a candidate gene case-control study for spontaneous PTD [21] using a case-parent triad design, which includes information from the paternal genome, and a hybrid design combining case-parent triads and control-mother dyads. Few studies have used either of these designs for PTD and none have done so in combination. These approaches provide several advantages over case-control designs in terms of minimizing potential population stratification (case-parent triad design) and their ability to increase study power (hybrid design) [22,23].

In our study we included the analysis of haplotypes. Haplotypes are in some cases preferable to SNPs, because haplotypes can sometimes capture un-genotyped functional SNPs better than single SNP analyses [24]. We considered fetal and maternal effects separately and in combination. Finally, we examined the distribution of associating variants based on the KEGG pathways in which they exist, to see if particular pathways are over-represented in our associations, thereby providing more biological insight that would not be possible by focusing solely on single genes or SNPs.

## Methods

### Participants

In a recent case-control candidate genetic association study, fetal and maternal samples from the Norwegian Mother and Child Cohort Study (MoBa) were genotyped at 1,430 SNPs in 140 genes to association with spontaneous PTD [21]. In the current study the same data was used from case and control mother-infant dyads with the addition of paternal samples from case pregnancies. The Norwegian Mother and Child Cohort Study (MoBa) is a pregnancy cohort consisting of more than 107 000 pregnancies recruited from 1999-2008 [25]. The majority of all pregnant women in Norway were invited to participate through a postal invitation in connection with routine ultrasound examination at 17-18 weeks of gestation http://www.fhi.no/morogbarn. The participation rate was around 44% and a written informed consent was obtained from each participant. The MoBa study collected biological specimens from mother, father and offspring and data from questionnaires given to the mother and father. The study is linked to the Medical Birth Registry of Norway (MBRN). MBRN receives medical records from every birth that takes place in Norway after gestational week 16 (after 2002 data is from week 12) [26], and all records from this registry are included in the MoBa study database. In our analyses we used samples derived from Version 2 of the MoBa cohort that included 53,711 pregnancies.

Blood samples were collected from the mother and father at the ultrasound screening appointment at the 17^th^-18^th ^week of gestation [27]. A new blood sample from the mother and a cord blood sample from the child were drawn at delivery. The majority of samples were received at the MoBa Biobank the day after collection and DNA was extracted on the day of receipt as previously described [27].

Selection of cases and controls has been previously described [21]. Briefly, cases were defined as live, singleton spontaneous PTD between 154 and 258 days of gestation (22^0/7^-36^6/7 ^weeks) in women aged 20 to 34 years. No exclusion criteria were made for the fathers. Extracted DNA had to be available from the Biobank for both the mother and child for the family to be included. Extracted DNA also had to be available for the case fathers, but not for the controls. Controls were selected according to the same criteria as cases, except for gestational age that was between 273 and 286 days (39^0/7^and 40^6/7 ^weeks). Two hundred fifteen control dyads were randomly selected from the eligible dyads. Cases and controls were not matched on any variables. In Version 2 of the MoBa database we identified 203 case-parent triads eligible for the study. Among the case-parent triads, 9 of the fathers did not have available DNA and only the case-mother dyads were used.

### Candidate genes, SNP selection and genotyping

Selection of candidate genes was based on previous associations of maternal and fetal genes with spontaneous PTD and are described elsewhere [21]. A total of 1,536 SNPs were selected from 143 candidate genes, but ambiguous placement using the SNPper database http://snpper.chip.org assigned them to 167 genes; the analyses were done using this annotation. Genotyping was performed on the Illumina GoldenGate Assay system http://www.illumina.com/technology/goldengate_genotyping_assay.ilmn.

### Data pre-processing

Call-rate, deviations from Hardy-Weinberg equilibrium (HWE) and Mendelian inconsistencies were determined with PLINK http://pngu.mgh.harvard.edu/purcell/plink/[28]. Minor allele frequency (MAF) calculations and additional analyses were performed using HAPLIN http://www.uib.no/smis/gjessing/genetics/software/haplin/[22,29,30]. Of the selected SNPs, 1443 SNPs were successfully genotyped with call-rates greater than 90%. Of these a total of 31 SNPs on the X-chromosome, 18 SNPs that deviated from HWE (p < 0.01) in controls and 68 SNPs with a minor allele frequency of < 5% were excluded from analyses, leaving 1,326 SNPs within 159 genes (Additional file 1 Table S1). Pedigrees assessed for Mendelian inconsistencies were removed if more than 1% of the SNPs showed evidence of such; two case triads and three control dyads were removed based on this criterion. In addition, families were excluded if the mother or the offspring had low call-rates (< 95%). If the father had low call-rate, data from his DNA was excluded from analysis, but the rest of the family remained in the study. The final sample size consisted of 407 fetal samples (196 cases, 211 controls), 407 maternal samples (196 cases, 211 controls) and 186 paternal samples (cases only).

### Data analysis

The single locus associations and the haplotype analyses were performed using HAPLIN software. Haplin can analyze case-parent triad data, case-control data and hybrid designs combining data from both case triads and control triads. It uses a full likelihood model, and estimates both population frequencies and relative risks relating to each haplotype [29]. The case-parent triad design has advantages and disadvantages relative to the case-control design [31]. For example, population-based case-control designs may be affected by population stratification, while family-based designs are robust to this [31]. Case-parent triad analyses and hybrid analyses also make it possible to better evaluate the balance of maternal and fetal effects. This is a substantial advantage for phenotypes that have their origins in fetal life and therefore can be influenced by both maternal genetics and the intra-uterine environment [32]. Simply comparing case mothers with control mothers or case children with control children does not account for differential effects of maternal and fetal genotypes. Triad analysis assumes mating symmetry in the population at large, and estimates the effects of maternal and fetal genes simultaneously. However, case-parent triad analyses have slightly less power than case-control studies and cannot estimate exposure effects [31]. The hybrid design combines case-parent triads and control-mother dyads in a joint likelihood model, and thus has a higher power than the case-parent triad and the case-control designs used separately [23,33]. Hybrid analyses may, however, still be vulnerable to the effects of population stratification, though less so than the case-control design [23]. Therefore, we have re-analyzed data from a previous study to assess if: 1) we find evidence for association in the same genes as previously reports, and 2) if new genes can be detected using this family based analysis plan.

SNPs and haplotypes were analyzed using the case-parent triad design and a hybrid design combining case-parent triads and control-mother dyads. The analyses were done both by looking at the effect of the fetal genes alone and by combining the effects of fetal and maternal alleles to avoid confounding by maternal genes [34]. In the combined estimation model, separate relative risks for fetal and maternal effects are estimated simultaneously in a joint model, adjusted for each other. The combined p-value refers to a likelihood ratio test comparing a full model including fetal to maternal effects with a null model with no effects whatsoever. In addition, we performed Wald tests to assess whether a second genome contributed significantly to PTD relative to only a single genetic contribution.

In addition to calculating p-values for individual SNPs, haplotypes were analyzed using overlapping sliding-windows of three SNPs. Haplotype significance and effect sizes were calculated relative to the most frequent haplotype. A multiplicative gene-dose model was assumed. To control for multiple testing within a gene, a single overall p-value was computed for each gene, using a score test procedure in Haplin [35]. To assess the effect of multiple testing as a whole, QQ-plots were used to plot the observed p-values against p-values expected purely by chance, i.e., p-values drawn from a uniform distribution.

Pathway analyses were performed using R http://www.r-project.org/[36]. The pathway analysis aimed at identifying pathways whose genes taken together are more associated with disease than random candidate genes from our study. That is, the criterion for significance of a pathway is that it has more genes associating with PTD than the "background" effects from our candidate genes as opposed to an *a priori *statistical distribution. This is a more conservative than the null hypothesis of no effects of any of the included genes. Pathways were analyzed using results of case-parent triads and a hybrid design using both case-parent triads and control-mother dyads. The analyses were done using fetal SNPs alone as well as using a combined estimate of fetal and maternal SNPs. Adjusted p-values for genes were matched to respective KEGG pathways using the KEGG_2_snp_b129 annotation http://www.genome.jp/kegg/[37]. Combined pathway-specific p-values were then obtained using a Fisher combination of p-values. That is, the combined p-value for a pathway is computed from a Chi-squared distribution with 2 k degrees of freedom, using -2(log(p_1_) +... +log(p_k_)) as the test statistic, where k is the number of genes in the pathway and p_i _is the p-value for gene in the pathway. The Fisher combination of p-values assumes independence between genes within the same pathway, which may not strictly be the case. We performed 10,000 simulations where the test statistic for a pathway was compared to the simulated test statistics obtained from drawing genes randomly from our study, each time selecting the same number of genes as found in the specific pathway. The resulting simulated pathway p-values were practically identical to the Fisher chi-squared values. In total 212 pathways were assessed (Additional file 2 Table S2).

### Ethics approval

Approval for this study was obtained from the Regional Committee for Medical Research and Ethics (S-06075) and the Norwegian Data Inspectorate (05/016784).

## Results

As expected from the case definition, cases and controls differed with respect to gestational age and birth weight (Table 1). In addition, there were significantly more primiparous women and women with a previous PTD in the case group than in the control group (Table 1). No other demographic differences existed between cases and controls.

**Table 1:** Clinical and demographical characteristics

	Cases (n = 196)	Controls (n = 211)	p
Maternal age (years)	29 [20-34]	30 [21-34]	0.135
Gestational age (days)	253 [172-258]	280 [273-286]	< 0.001
Primiparity	128 (65.3%)	98 (46.4%)	< 0.001
Smoking wk 1-17 of pregnancy (%)	64 (32.7%)	55 (26.1%)	0.124
Pre-pregnancy BMI	23.03 [14.84-41.26]	23.23 [17.30-38.10]	0.561
Birth weight (g)	2815 [747-4000]	3645 [2610-4970]	< 0.001
Gender infant, male	51.5%	42.7%	0.073
Paternal age (years)	31 [20-46]	31 [23-48]	0.561
Previous PTD	18 (9.2%)	3 (1.4%)	< 0.001
Gestational bleeding, 2^nd ^trimester	28 (15.8%)	21 (10.9%)	0.161

^1 ^Medians are reported with the range in brackets.

^2 ^Cases are defined as preterm delivery < 37 weeks of gestation, while controls are defined as term delivery from 39+0 to 40+6 weeks of gestation.

^3 ^P-values are calculated by Mann-Whitney U test for continuous variables and chi-square test for categorical variables.

^4 ^Information about pre-pregnancy BMI and smoking in week 1-17 of pregnancy is self reported and collected from MoBa Questionnaire 1.

### Analysis of fetal genes

**Figure 1:**
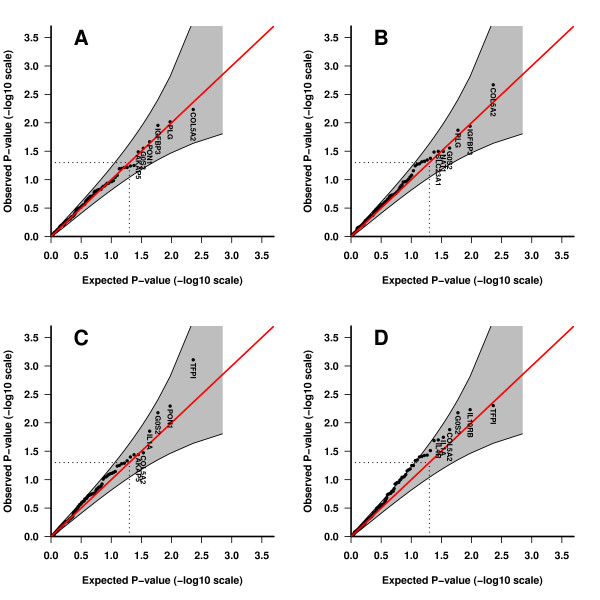
**Fetal results**. QQ-plots showing the most significant fetal genes for the different designs. A) Single locus, case-parent triad. B) Haplotype, case-parent triad. C) Single locus, hybrid. D) Haplotype, hybrid. The observed p-values for each gene are plotted against the expected p-values. If there is no effect of the genes, the p-values will be positioned on the straight line. The grey area along the straight line shows the point-wise confidence interval for the expected p-values. Correction for multiple testing was performed within each gene. There have been done no adjustments for covariates in the hybrid analysis.

Significant associations were observed in the fetal analyses. The most significant gene in the case-parent triad approach was COL5A2 (collagen V alpha-2) with p = 0.006 in the single locus analysis and p = 0.002 in the haplotype analysis (Table 2 Figure 1). This gene was also significant in the hybrid analysis (Table 2 Figure 1). Within this gene several SNPs showed evidence of association (Table 3 and 4), as were several haplotypes (Table 5 and 6).

**Table 2:** Significant fetal genes

	Case-parent triad design		Hybrid design	
	Single locus	Haplotype	Single locus	Haplotype
COL5A2	**0.006**	**0.002**	**0.034**	**0.013**
PLG	**0.010**	**0.014**	0.336	0.530
IGFBP3	**0.011**	**-**	0.091	-
PON1	**0.022**	0.053	**0.005**	**0.021**
G0S2	**0.028**	**-**	**0.007**	**-**
AKAP5	**0.032**	**-**	**0.036**	**-**
PTCRA	0.061	**0.042**	0.098	0.112
IL1A	0.064	0.090	**0.014**	**0.018**
SMCR8	0.084	-	**0.040**	**-**
SLC23A1	0.091	**0.032**	0.336	0.091
TCN2	0.130	-	**0.036**	**-**
TFPI	0.131	0.317	**0.001**	**0.005**
IL4	0.150	0.186	**0.047**	**0.045**
DEFA3	0.152	**0.047**	0.146	0.070
CRH	0.157	0.269	0.072	**0.031**
IL10RB	0.275	0.193	0.156	**0.006**
NAT1	0.285	**0.032**	0.546	0.118
MMP8	0.319	0.278	0.216	**0.046**
CD14	0.328	**0.049**	0.616	0.309
TREM1	0.387	0.224	0.189	**0.039**
TNFRSF1B	0.770	**0.045**	0.458	0.161
IL4R	0.814	0.277	0.079	**0.020**

^1 ^Correction for multiple testing has been performed within each gene.

**Table 3:** Significant SNPs in COL5A2 (collagen V alpha-2) and COL5A1 (collagen V alpha-1)

				Fetal			Maternal			Combined
gene	snp	allele	MAF	RR	95% CI	p	RR	95% CI	p	overall p
COL5A2	rs3923384	G	0.034	2.29	1.12, 4.54	**0.021**	0.83	0.43, 1.59	0.568	**0.048**
	rs6434322	A	0.034	2.29	1.12, 4.54	**0.021**	0.83	0.43, 1.59	0.568	**0.048**
	rs10165260	G	0.034	2.29	1.12, 4.54	**0.021**	0.83	0.43, 1.59	0.568	**0.048**
	rs7420331	G	0.154	0.47	0.30, 0.73	**0.001**	1.09	0.70, 1.69	0.710	**0.003**
COL5A1	rs4842161	C	0.482	1.32	0.99, 1.75	0.060	0.75	0.57, 1.00	0.055	**0.024**
	rs3124932	A	0.433	1.53	1.14, 2.03	**0.005**	0.78	0.58, 1.04	0.084	**0.003**
	rs12005720	G	0.137	0.83	0.56, 1.22	0.343	1.61	1.08, 2.40	**0.018**	**0.034**
	rs3128621	A	0.440	1.49	1.12, 1.99	**0.008**	0.70	0.53, 0.93	**0.015**	**0.001**
	rs4842167	G	0.388	1.51	1.13, 2.03	**0.007**	0.70	0.52, 0.93	**0.016**	**0.001**
	rs3811161	G	0.509	0.70	0.52, 0.93	**0.012**	1.32	0.99, 1.75	0.060	**0.007**
	rs3811152	G	0.092	0.59	0.36, 0.95	**0.032**	1.72	1.05, 2.79	**0.031**	**0.008**
	rs10745387	A	0.405	1.36	1.02, 1.82	**0.037**	0.75	0.56, 1.00	0.055	**0.016**

Case-parent triad analysis

^1 ^Results are not corrected for multiple testing.

^2 ^A multiplicative model is assumed.

**Table 4:** Significant SNPs in COL5A2 (collagen V alpha-2) and COL5A1 (collagen V alpha-1)

				Fetal			Maternal			Combined
gene	snp	allele	MAF	RR	95% CI	p	RR	95% CI	p	overall p
COL5A2	rs3923384	G	0.042	1.95	1.14,3.31	**0.015**	0.76	0.42, 1.35	0.347	0.055
	rs6434322	A	0.042	1.94	1.14,3.31	**0.013**	0.76	0.42, 1.35	0.342	0.055
	rs10165260	G	0.042	1.95	1.14,3.31	**0.015**	0.76	0.42, 1.35	0.347	0.055
	rs7420331	G	0.129	0.53	0.35,0.81	**0.004**	1.28	0.87, 1.85	0.199	**0.008**
COL5A1	rs3124311	C	0.510	0.77	0.60, 0.99	**0.045**	1.06	0.83, 1.37	0.624	0.130
	rs4842157	G	0.364	0.75	0.57, 0.99	**0.039**	1.07	0.81, 1.39	0.631	0.115
	rs4842161	C	0.458	1.40	1.10, 1.80	**0.008**	0.81	0.63, 1.03	0.085	**0.017**
	rs3124932	A	0.433	1.52	1.19, 1.96	**0.0004**	0.78	0.61, 1.00	0.054	**0.002**
	rs12005720	G	0.161	0.74	0.53, 1.04	0.090	1.40	1.01, 1.94	**0.043**	0.063
	rs3128621	A	0.441	1.48	1.16, 1.90	**0.001**	0.70	0.54, 0.90	**0.006**	**0.001**
	rs4842167	G	0.424	1.37	1.07, 1.76	**0.014**	0.64	0.49, 0.82	**0.001**	**0.001**
	rs3811161	G	0.465	0.78	0.61, 1.00	0.055	1.49	1.16, 1.90	**0.003**	**0.004**
	rs3811152	G	0.103	0.55	0.35, 0.86	**0.009**	1.54	1.04, 2.27	**0.033**	**0.009**
	rs10745387	A	0.434	1.25	0.98, 1,61	0.073	0.70	0.54, 0.90	**0.005**	**0.011**

Hybrid analysis

^1 ^Results are not corrected for multiple testing.

^2 ^A multiplicative model is assumed.

**Table 5:** Fetal and maternal haplotypes in COL5A2

			Fetal haplotypes			Maternal haplotypes			Combined
Markers	Haplotype	freq	RR	95% CI	p	RR	95% CI	p	Overall p
rs6760780-rs3923384-rs6434317	T-A-C	0.810	1.00	-	-	1.00	-	-	0.006
	T-A-A	0.116	0.55	0.33, 0.92	0.021	0.97	0.59, 1.60	0.920	
	A-A-A	0.035	0.33	0.13, 0.85	0.020	1.50	0.64, 3.50	0.351	
	A-G-A^1^	0.034	2.11	1.05, 4.22	0.035	0.84	0.44, 1.60	0.594	
rs3923384-rs6434317-rs6434322	A-C-G	0.810	1.00	-	-	1.00	-	-	0.003
	A-A-G^1^	0.154	0.49	0.31, 0.77	0.002	1.08	0.70, 1.67	0.724	
	G-A-A	0.034	2.09	1.03, 4.19	0.042	0.83	0.44, 1.60	0.588	
rs6434317-rs6434322-rs10165260	C-G-A	0.810	1.00	-	-	1.00	-	-	0.003
	A-G-A^1^	0.154	0.49	0.31, 0.76	0.002	1.07	0.69, 1.65	0.747	
	A-A-G	0.034	2.09	1.03, 4.18	0.040	0.83	0.43, 1.60	0.584	
rs6434322-rs10165260-rs7420331	G-A-A	0.810	1.00	-	-	1.00	-	-	0.003
	G-A-G	0.154	0.49	0.31, 0.77	0.001	1.08	0.70, 1.67	0.726	
	A-G-A	0.035	2.09	1.03, 4.19	0.041	0.83	0.44, 1.61	0.592	
rs10165260-rs7420331-rs13024858	A-A-A	0.762	1.00	-	-	1.00	-	-	0.010
	A-G-A	0.149	0.50	0.31, 0.79	0.003	1.00	0.64, 1.58	0.993	
	A-A-C	0.047	1.20	0.58, 2.44	0.631	0.57	0.27, 1.20	0.138	
	G-A-A	0.028	1.74	0.72, 4,24	0.211	0.50	0.21, 1.19	0.122	
	G-A-C	0.008	2.37	0.71, 7.54	0.151	1.70	0.56, 5.11	0.343	
rs7420331-rs13024858-rs6752781	A-A-A	0.794	1.00	-	-	1.00	-	-	0.016
	C-A-A	0.151	0.48	0.30, 0.77	0.002	1.03	0.65, 1.60	0.913	
	A-C-G	0.053	1.43	0.77, 2.63	0.259	0.81	0.44, 1.48	0.492	

Case-parent triad analysis

^1 ^Haplotype deviates from Hardy-Weinberg equilibrium.

^2 ^Calculation of haplotypes has been performed in Haplin with a sliding window of three SNPs.

^3 ^Haplotypes are not adjusted for multiple testing.

**Table 6:** Fetal and maternal haplotypes in COL5A2

			Fetal haplotypes			Maternal haplotypes			Combined
markers	haplotype	freq	RR	95% CI	p	RR	95% CI	p	Overall p
rs6760780-rs3923384-rs6434317	T-A-C	0.830	1.00	-	-	1.00	-	-	0.013
	T-A-A	0.091	0.65	0.41, 1.05	0.077	1.20	0.77, 1.86	0.419	
	A-G-A	0.041	1.82	1.06, 3.08	0.030	0.78	0.44, 1.41	0.413	
	A-A-A	0.037	0.33	0.14, 0.81	0.014	1.48	0.76, 2.88	0.244	
rs3923384-rs6434317-rs6434322	A-C-G	0.828	1.00	-	-	1.00	-	-	0.006
	A-A-G	0.129	0.54	0.36, 0.82	0.004	1.26	0.87, 1.84	0.241	
	G-A-A	0.042	1.81	1.06, 3.10	0.029	0.78	0.43, 1.39	0.393	
rs6434317-rs6434322-rs10165260	C-G-A	0.829	1.00	-	-	1.00	-	-	0.006
	A-G-A	0.129	0.55	0.36, 0.83	0.006	1.27	0.88, 1.84	0.210	
	A-A-G	0.042	1.81	1.06, 3.10	0.026	0.77	0.43, 1.40	0.385	
rs6434322-rs10165260-rs7420331	G-A-A	0.829	1.00	-	-	1.00	-	-	0.006
	G-A-G	0.129	0.54	0.36, 0.82	0.004	1.26	0.87, 1.83	0.232	
	A-G-A	0.042	1.80	1.06, 3.11	0.026	0.78	0.43, 1.39	0.392	
rs10165260-rs7420331-rs13024858	A-A-A	0.792	1.00	-	-	1.00	-	-	0.041
	A-G-A	0.109	0.58	0.37, 0.91	0.018	1.33	0.90, 1.97	0.159	
	A-A-C	0.035	1.46	0.78, 2.76	0.242	0.70	0.35, 1.44	0.329	
	G-A-A	0.022	2.23	1.12, 4.57	0.023	0.63	0.28, 1.40	0.262	
	A-G-C	0.020	0.38	0.10, 1.55	0.175	0.72	0.23, 2.29	0.582	
	G-A-C	0.019	1.44	0.65, 3.20	0.362	0.95	0.42, 2.23	0.901	
rs7420331-rs13024858-rs6752781	A-A-A	0.817	1.00	-	-	1.00	-	-	0.043
	G-A-A	0.109	0.55	0.35, 0.86	0.009	1.37	0.92, 2.05	0.116	
	A-C-G	0.053	1.41	0.86, 2.38	0.187	0.83	0.48, 1.42	0.516	
	G-C-G	0.020	0.47	0.13, 1.81	0.277	0.70	0.22, 2.22	0.542	

Hybrid analysis

^1 ^Calculation of haplotypes has been performed in Haplin with a sliding window of three SNPs.

^2 ^Haplotypes are not adjusted for multiple testing.

The most significant single locus association was with the G allele at rs7420331. This SNP had a p-value of 0.001 with a relative risk (RR) of 0.47 (confidence interval, (CI): 0.30, 0.73) in the case-parent triad analysis and a p-value of 0.004 and a RR of 0.53 (CI: 0.35, 0.85) in the hybrid analysis, indicating that the G allele protects against spontaneous PTD. The single locus association, rs7420331, also had a significant uncorrected genotypic result in the previous case-control study (p = 0.01) [21]. The other three SNPs in COL5A2 had a p-value of 0.021 and an RR of 2.29 (CI: 1.12, 4.54) in the case-parent triad analysis. In the hybrid analysis the p-value was 0.015 with an RR of 1.95 (CI: 1.14, 3.31). This indicates that these SNPs associate with increased risk of spontaneous PTD, but since they are in strong linkage disequilibrium with each other they cannot be considered independently and most likely tag a single causal variant. In the hybrid analysis the most significant gene was TFPI (tissue factor pathway inhibitor), which also was the most significant fetal gene in the previous case-control study on the same samples [21]. However, this gene was not significant in the case-parent triad analysis, except for one SNP at rs6434222. PON1 (paraoxonase 1) was significant using all analytical approaches, except for the haplotype analysis in the case-parent triad design where it was borderline significant (p = 0.053) (Table 2). Moreover, this gene was found to be significant in fetal samples in the previous published case-control analysis [21]. The most significant SNP in this gene was rs854552 for all three approaches, with the G allele conferring a protective effect against PTD (p = 0.001 in the case-parent triad analysis and p = 0.0003 in the hybrid analysis).

The three haplotypes in COL5A2 that were the most significant were equivalent in their association with PTD: G-A-G at rs6434322-rs10165260-rs7420331 (p = 0.001) with an RR of 0.49 (CI: 0.31, 0.77), A-A-G at rs3923384-rs6434317-rs6434322 (RR = 0.49, CI: 0.31, 0.77) and A-G-A at rs6434317-rs6434322-rs10165260 (RR = 0.49, CI: 0.31, 0.77) (Table 7). This indicates that they tag the same associating variant(s).

**Table 7:** Significant genes when maternal and fetal alleles are combined

	Case-parent triad design		Hybrid design	
	Single locus	Haplotype	Single locus	Haplotype
IGFBP3	**0.010**	**-**	0.216	-
GSTP1	**0.014**	**-**	**0.016**	**-**
COL5A1	**0.018**	0.104	**0.014**	0.077
COL5A2	**0.022**	**0.016**	0.059	**0.031**
MTHFD1	**0.023**	0.051	**0.048**	0.116
PLG	**0.025**	**0.049**	0.121	0.144
MMP8	**0.034**	0.073	**0.041**	**0.008**
IL10RA	0.050	**0.028**	0.083	**0.044**
SLC23A1	0.066	**0.007**	0.549	0.215
PON1	0.082	0.131	**0.025**	0.120
G0S2	0.085	-	**0.022**	**-**
IL1A	0.187	0.276	**0.048**	0.085
TFPI	0.304	0.599	**0.004**	**0.039**
SERPINH1	0.354	0.088	0.216	**0.016**
TREM1	0.459	0.268	0.106	**0.041**
SLC6A4	0.525	0.346	**0.032**	**0.045**
HSPA6	0.583	0.310	**0.050**	0.182
IL10RB	0.618	0.584	0.406	**0.031**

^1 ^Correction for multiple testing has been performed within each gene.

### Combined analysis of fetal and maternal genes

**Figure 2:**
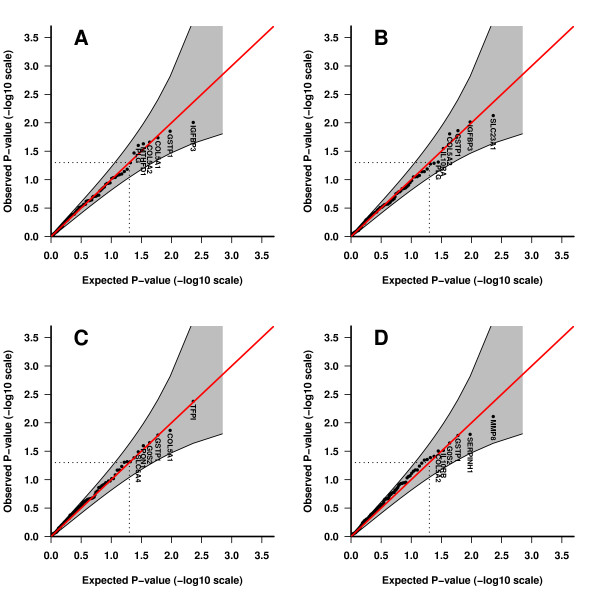
**Combined results for fetal and maternal genes**. QQ-plots showing the most significant results for the different designs when the effects of maternal and fetal genes are combined. A) Single locus, case-parent triad. B) Haplotype, case-parent triad. C) Single locus, hybrid. D) Haplotype, hybrid.

When including maternal effects COL5A2 remained significant in all analyses except the hybrid single locus approach, which was borderline (p = 0.059) (Table 7 Figure 2). In addition a related gene, COL5A1 (collagen V alpha-1), was significant in the single locus analysis both in the case-parent triad approach and in the hybrid approach. When looking at maternal and fetal SNPs in COL5A2 separately (Table 3 and 4), it is evident that the SNPs were significant only for the fetal genotypes, but not the maternal. The combined effect of the fetal and the maternal genotypes is less significant than the fetal gene, indicating that the association with this gene is driven by the fetal genome. The same was true for the haplotypes (Table 5 and 6). COL5A1 on the other hand, showed significance for both fetal and maternal genotypes, but the overall p-value for the gene did not reach significance when considering fetal SNPs alone. When looking at the significant fetal and maternal SNPs in COL5A1 separately and combined it becomes clear that the combined effect for several of the fetal and maternal SNPs are stronger than when considered separately. This implies a combined effect of fetal and maternal alleles. In the case-control analysis [21] one fetal SNP in COL5A2 (rs7420331) and one in COL5A1 were significant in the uncorrected analysis. In the maternal samples, five SNPs were significant in COL5A1. The most significant gene in the hybrid single locus analysis was TFPI (tissue factor plasminogen inhibitor) (Table 2, 5 and 6). In the haplotype analysis the most significant genes were SLC23A1 (Solute carrier family 23 member 1) for the case-parent triad approach and MMP8 (matrix metalloproteinase 8) for the hybrid approach (Table 2).

### Pathway analysis

We identified several pathways as significantly associating with spontaneous PTD. The most significant fetal pathways were Focal Adhesion (hsa04510), p53 signaling (hsa04115), Cell Communication (hsa01430), and ECM (extracellular matrix) receptor interaction (hsa04512) (Table 8). Looking at the combined effect of maternal and fetal SNPs the most significant pathways were Glutathione metabolism (hsa00480) and Prostate Cancer (hsa05215) (Table 9). Cell Communication, ECM-receptor interaction and Focal Adhesion remained significant when including maternal effects.

**Table 8:** Significant fetal pathways in spontaneous preterm delivery

		Case-parent triad		Hybrid	
KEGG pathway	Pathway Annotation	Single locus	Haplotype	Single locus	Haplotype
hsa04510	Focal Adhesion	**0.017**	**0.019**	0.103	**0.016**
hsa04115	p53 signaling pathway	**0.022**	**0.010**	0.054	0.087
hsa01430	Cell Communication	**0.024**	**0.020**	0.107	**0.012**
hsa04512	ECM-receptor interaction	**0.024**	**0.020**	0.107	**0.012**
hsa00361	Gamma-Hexachlorocyclohexane	0.059	0.143	**0.016**	**0.030**
hsa00363	Bisphenol A degradation	0.059	0.143	**0.016**	**0.030**
hsa04330	Notch signaling pathway	0.061	**0.042**	0.098	0.112
hsa04210	Apoptosis	0.114	0.211	**0.027**	0.127
hsa00680	Methane metabolism	0.194	0.261	**0.022**	**0.022**
hsa04640	Hematopoietic cell lineage	0.438	0.317	0.083	**0.040**
hsa04060	Cytokine-cytokine receptor interaction	0.500	0.210	0.080	**0.033**
hsa04630	Jak-STAT signaling pathway	0.522	0.197	**0.043**	**0.013**

**Table 9:** Significant pathways when the effects of maternal and fetal genes are combined

		Case-parent triad		Hybrid	
KEGG pathway	Pathway Annotation	Single locus	Haplotype	Single locus	Haplotype
hsa00480	Gluthatione metabolism	**0.014**	**0.014**	**0.016**	**0.017**
hsa05215	Prostate Cancer	**0.017**	**0.023**	**0.013**	**0.026**
hsa00630	Glyoxylate and dicarboxylate metabolism	**0.023**	0.051	**0.048**	0.116
hsa01430	Cell Communication	**0.025**	0.081	**0.029**	**0.010**
hsa04512	ECM receptor interaction	**0.025**	0.081	**0.029**	**0.010**
hsa04510	Focal Adhesion	**0.030**	0.116	0.061	**0.025**
hsa00361	Gamma Hexachlorocyclohexane	**0.036**	0.084	**0.044**	0.145
hsa00363	Bisphenol A degradation	**0.036**	0.084	**0.044**	0.145

## Discussion

In the present study we presented a re-analysis of previously published data that further elucidated the relative roles of maternal and fetal genomes on spontaneous PTD. In the previous study that used overlapping data, the most significantly associated genes were COL1A2 and PTGER3 in the maternal and TFPI and PON1 in the fetal analyses. We confirmed in our analyses an association with PON1. However, TFPI, which was found in the previous study, was only significant in our hybrid analysis. It is likely that the original finding was due to population stratification, a factor minimized by the family based analyses we used.

Using our approach, the most consistent significant gene across all analyses was COL5A2, which is involved in the production of type V collagen. The previous analysis only provided minimal evidence for the association with this gene [21]. COL5A1, which also contributes to the production of type V collagen, was also found to be significant in the single locus analysis when maternal effects were included, and several SNPs were significant when examining maternal and fetal alleles separately (Table 5 and 6). Type V collagen plays a critical role in early fibril initiation and in the determination of fibril structure and matrix organization [38]. Defects in type V collagen due to mutations in COL5A1 and COL5A2 are the cause of the classical type (types I and II) of the heritable connective tissue disorder Ehler-Danlos syndrome that confers an increased risk for PTD if the fetus is affected, especially from pPROM [39-41]. It is therefore reasonable to hypothesize that variations in these genes might be involved in the pathophysiology leading to PTD. However, the results must be interpreted with care, as the QQ-plots shows that the observed p-values do not deviate from what would be expected by chance. Few other studies have tested the association between COL5A2 and the risk of spontaneous PTD. A recent study by Romero et al found an association between rs189683203 in fetal DNA and the risk of pPROM with an unadjusted p-value of 0.021 (odds ratio, (OR) = 1.42, CI: 1.06, 1.92) [17]. In another study on the same study population the authors found an association between rs6750027 in maternal DNA and the risk of PTL with an unadjusted p-value of 0.043 (OR = 1.32, CI: 1.01, 1.74) [13]. Another study by Velez et al found significant associations in three fetal SNPs and one maternal SNP in COL5A2 [12].

PON1 was significantly associated with PTD in both the triad analysis and the hybrid analysis of the fetal genes alone, but not in the combined analysis of maternal and fetal genes. This is most likely because the combined effects of maternal and fetal genes may not reach significance due to reduced power in this type of analysis. None of the maternal SNPs showed an association with spontaneous PTD, while several of the fetal SNPs did. PON1 was found to be significantly associated with PTD in the previously published case-control analysis on the same fetal samples as well, and possible mechanisms of how this gene might contribute to preterm delivery are discussed there [21].

TFPI which was the strongest fetal association in the case-control study [21], showed at best weak association in the triad-analysis, but was the most significant gene in the hybrid design. The most significant SNP in the case-control study, rs6434222 in TFPI, also had a significant unadjusted p-value in the triad analysis (p = 0.02).

Overall, the results from the case-parent-triad analysis and the previously performed case-control analysis only overlapped in a few genes. The results from the hybrid analysis lay somewhere in-between the results from the case-parent triad and the case-control study. These differences may indicate that within our study population there was population stratification that could have led to spurious results in the case-control analysis. This is minimized using triads. Although the hybrid analysis has more power than the triad analysis, it may still be affected by population stratification, but to a lesser degree than a case-control design. The case-parent triad analysis is therefore the most reliable in terms of reducing the problem of stratification, and we present these as our most compelling results.

Our study also identified several pathways as associating with PTD. Significant results were found in the Focal Adhesion, Cell Communication and ECM-receptor interaction pathways, all of which include COL5A2 and COL5A1, but none of the other associated genes in our study. The ECM-receptor interaction pathway is involved in tissue and organ morphogenesis associated with the bleeding disorders Bernard-Soulier syndrome and Glanzmann thrombasthenia. The Focal adhesion pathway is involved in cell matrix adhesion and also associated with the bleeding disorder Glanzmann thrombasthenia. For the Notch signaling (hsa04330), Gluthatione Metabolism (hsa00480) and Glyoxylate and dicarboxylate metabolism (hsa00630) pathways only one gene was available for inclusion and the results from these pathways must be interpreted with care. Because this was a candidate gene study, the number of included genes and SNPs in each pathway was limited. Nevertheless, those pathways that provide strong evidence of association can probably be taken as truly being involved in spontaneous PTD.

The major strength of this study was that we used the case-parent triad design and the hybrid design and compared these results to those of the traditional case-control design. Few other candidate gene studies on PTD have been performed using the case-triad design, which offers protection against bias due to population stratification. Additionally, we performed a hybrid analysis, which has increased statistical power over both the case-triad and the case-control designs. These designs also provide separate estimates of fetal and maternal alleles, as well as an overall p-value estimating the combined effect of maternal and fetal alleles. In this way, confounding through maternal alleles, which can affect the intrauterine environment and thus the phenotype of the fetus, can be avoided. Our study was limited in that the small sample size was small compared to modern GWAS level analyses and in that it was based on a limited number of candidate genes. Also, no covariates were included in the hybrid analysis and we were not able to separate spontaneous PTD into pPROM and PTL at the time of analysis. Another weakness of this study is that we did not have an external replication sample to corroborate our findings. The findings should thus be regarded as exploratory, although the prior plausibility of the genes provides increased confidence in our results.

## Conclusion

The results from this study suggest that fetal SNPs and haplotypes in COL5A2 and a combined effect of fetal and maternal SNPs in COL5A1 are associated with spontaneous PTD. The significant pathways, Focal adhesion, Cell communication and ECM receptor interaction all included COL5A2 and COL5A1.

## Competing interests

The authors declare that they have no competing interests.

## Authors' contributions

All authors planned the study and contributed with interpretation of results and writing of the paper. SM, RM, HKG, SMW, KKR and BJ designed the study and SM, RM, NHM and BJ selected cases and controls. SM, RM and HKG analyzed the data. All authors have read and approved the final manuscript.

## Pre-publication history

The pre-publication history for this paper can be accessed here:

http://www.biomedcentral.com/1471-2350/12/174/prepub

## Supplementary Material

Additional file 1 **Table S1 Single-nucleotide polymorphisms and genes examined**. Table showing which single-nucleotide polymorphisms and genes that have been examined in the study. Click here for file

Additional file 2 **Table S2 KEGG pathways examined**. Table showing which KEGG pathways that have been examined in the study. Click here for file
