# Supplementary material for: Candidate gene analysis of spontaneous preterm delivery: New insights from re-analysis of a case-control study using case-parent triads and control-mother dyads
Source: BMC Med Genet. 2011 Dec 30;12:174. doi: 10.1186/1471-2350-12-174 (PMC3260094; doi:10.1186/1471-2350-12-174)
Supplement: Additional file 1 — Table S1 Single-nucleotide polymorphisms and genes examined. Table showing which single-nucleotide polymorphisms and genes that have been examined in the study. [file 1471-2350-12-174-S1.DOC]

**Table S1. Single-nucleotide polymorphisms and genes examined.**

| Gene | SNP |
| --- | --- |
| PAFAH1B1 | rs6502385 |
| PAFAH1B1 | rs7209407 |
| PAFAH1B1 | rs7213463 |
| PAFAH1B1 | rs3785958 |
| PAFAH1B1 | rs1029744 |
| PAFAH1B1 | rs11078302 |
| PAFAH1B1 | rs12938775 |
| PAFAH1B1 | rs2317297 |
| PAFAH1B1 | rs7223411 |
| PAFAH1B1 | rs4790353 |
| KIAA0664 | rs4790355 |
| KIAA0664 | rs4790356 |
| IL2RA | rs10795737 |
| IL2RA | rs12359875 |
| IL2RA | rs12722605 |
| IL2RA | rs12244380 |
| IL2RA | rs9663421 |
| IL2RA | rs12722596 |
| IL2RA | rs2386841 |
| IL2RA | rs12722588 |
| IL2RA | rs2076846 |
| IL2RA | rs7093069 |
| IL2RA | rs2031229 |
| IL2RA | rs12722563 |
| IL2RA | rs7910961 |
| IL2RA | rs6602392 |
| IL2RA | rs7072398 |
| IL2RA | rs11256457 |
| IL2RA | rs6602398 |
| IL2RA | rs11598648 |
| IL2RA | rs4749926 |
| IL2RA | rs1107345 |
| IL2RA | rs11256497 |
| IL2RA | rs791589 |
| IL2RA | rs791590 |
| IL2RA | rs2476491 |
| IL2RA | rs706779 |
| IL2RA | rs706778 |
| IL2RA | rs3134883 |
| IL2RA | rs7072793 |
| IL2RA | rs7073236 |
| PLEKHG6 | rs740841 |
| PLEKHG6 | rs2302350 |
| TNFRSF1A | rs1800693 |
| TNFRSF1A | rs1860545 |
| TNFRSF1A | rs4149578 |
| TNFRSF1A | rs4149577 |
| TNFRSF1A | rs4149570 |
| SCNN1A | rs3764874 |
| DEFA3 | rs17466573 |
| DEFA3 | rs7825750 |
| MGC5297 | rs16879258 |
| MGC5297 | rs17184211 |
| MGC5297 | rs1801394 |
| MTRR | rs7730643 |
| MTRR | rs326121 |
| MTRR | rs326124 |
| MTRR | rs1532268 |
| MTRR | rs7703033 |
| MTRR | rs162031 |
| MTRR | rs162033 |
| MTRR | rs162036 |
| MTRR | rs3815743 |
| MTRR | rs10380 |
| MTRR | rs8659 |
| MTHFR | rs4846048 |
| MTHFR | rs4846049 |
| MTHFR | rs1476413 |
| MTHFR | rs1801131 |
| MTHFR | rs12121543 |
| MTHFR | rs1994798 |
| MTHFR | rs1801133 |
| MTHFR | rs17421462 |
| MTHFR | rs17421511 |
| MTHFR | rs4846052 |
| MTHFR | rs11121832 |
| MTHFR | rs9651118 |
| MTHFR | rs17367504 |
| MTHFR | rs3753582 |
| MTHFR | rs3737964 |
| TNFRSF1B | rs590368 |
| TNFRSF1B | rs652625 |
| TNFRSF1B | rs976881 |
| TNFRSF1B | rs3766730 |
| TNFRSF1B | rs616645 |
| TNFRSF1B | rs816050 |
| TIMP4 | rs3773364 |
| TIMP4 | rs4684841 |
| TNFRSF1B | rs474247 |
| TIMP4 | rs99365 |
| TNFRSF1B | rs1201157 |
| TNFRSF1B | rs1061622 |
| TIMP4 | rs3755724 |
| TNFRSF1B | rs5746051 |
| TNFRSF1B | rs5746053 |
| TNFRSF1B | rs235219 |
| TNFRSF1B | rs1061624 |
| TNFRSF1B | rs1061628 |
| TNFRSF1B | rs1061631 |
| TNFRSF1B | rs235214 |
| HSPA14 | rs10906772 |
| HSPA14 | rs11593057 |
| HSPA14 | rs17155992 |
| HSPA14 | rs7894284 |
| HSPA14 | rs9787671 |
| HSPA14 | rs7905174 |
| F2RL3 | rs2227356 |
| F2RL3 | rs773901 |
| F2RL3 | rs1054533 |
| F2RL3 | rs2608732 |
| NAT1 | rs10888150 |
| NAT1 | rs7017402 |
| NAT1 | rs4298522 |
| NAT1 | rs9325827 |
| NAT1 | rs17126350 |
| NAT1 | rs4921880 |
| NAT1 | rs7003890 |
| NAT1 | rs8190837 |
| NAT1 | rs8190870 |
| SMCR8 | rs1979277 |
| SHMT1 | rs2273027 |
| SHMT1 | rs2273026 |
| NAT2 | rs4646246 |
| NAT2 | rs7832071 |
| NAT2 | rs1801280 |
| NAT2 | rs1799929 |
| NAT2 | rs1208 |
| NAT2 | rs721398 |
| NAT2 | rs721399 |
| IL6 | rs1880243 |
| IL6 | rs12700386 |
| IL6 | rs1800797 |
| IL6 | rs1800795 |
| IL6 | rs2069840 |
| IL6 | rs1554606 |
| IL6 | rs11766273 |
| POMC | rs6713532 |
| SLC6A4 | rs7224199 |
| SLC6A4 | rs1042173 |
| SLC6A4 | rs3794808 |
| SLC6A4 | rs140701 |
| SLC6A4 | rs140700 |
| SLC6A4 | rs2020942 |
| SLC6A4 | rs6354 |
| SLC6A4 | rs2020936 |
| SLC6A4 | rs12150214 |
| SLC6A4 | rs4251417 |
| SLC6A4 | rs16965628 |
| SLC6A4 | rs2020933 |
| IL4R | rs2057768 |
| IL4R | rs6498012 |
| IL4R | rs4787948 |
| IL4R | rs3024530 |
| IL4R | rs3024537 |
| IL4R | rs3024547 |
| IL4R | rs3024548 |
| IL4R | rs3024560 |
| IL4R | rs2239347 |
| IL4R | rs3024623 |
| IL4R | rs4787423 |
| IL4R | rs3024676 |
| IL4R | rs2234898 |
| IL4R | rs1805015 |
| IL4R | rs8832 |
| IL4R | rs1029489 |
| IL4R | rs4787956 |
| EPHX2 | rs4149239 |
| EPHX2 | rs17057288 |
| EPHX2 | rs7816586 |
| EPHX2 | rs891401 |
| EPHX2 | rs721619 |
| EPHX2 | rs10503812 |
| EPHX2 | rs4149252 |
| EPHX2 | rs13269963 |
| EPHX2 | rs1042064 |
| EPHX2 | rs4149259 |
| EPHX2 | rs7341557 |
| EPHX2 | rs2640726 |
| TCN2 | rs2267163 |
| CCL2 | rs1024610 |
| CCL2 | rs3760396 |
| CCL2 | rs4586 |
| CCL2 | rs991804 |
| CCL8 | rs885691 |
| CCL8 | rs1233650 |
| CCL8 | rs3138034 |
| CCL8 | rs11575060 |
| CCL8 | rs3138039 |
| CCL8 | rs4794999 |
| CRHR2 | rs4722999 |
| CRHR2 | rs12701020 |
| CRHR2 | rs973002 |
| CRHR2 | rs929377 |
| CRHR2 | rs2240404 |
| CRHR2 | rs2190242 |
| CRHR2 | rs2284217 |
| CRHR2 | rs6462219 |
| CRHR2 | rs2284219 |
| CRHR2 | rs2267716 |
| CRHR2 | rs2267717 |
| CCL3 | rs1634491 |
| CCL3 | rs1851503 |
| CCL3 | rs9972960 |
| CCL3 | rs1634502 |
| TIMP3 | rs5754289 |
| TIMP3 | rs9606994 |
| TIMP3 | rs9619311 |
| TIMP3 | rs130274 |
| TIMP3 | rs11704261 |
| TIMP3 | rs738992 |
| TIMP3 | rs242089 |
| TIMP3 | rs130287 |
| TIMP3 | rs130290 |
| TIMP3 | rs130293 |
| TIMP3 | rs5749524 |
| TIMP3 | rs242082 |
| TIMP3 | rs80272 |
| TIMP3 | rs242078 |
| TIMP3 | rs242076 |
| TIMP3 | rs130300 |
| TIMP3 | rs242072 |
| TIMP3 | rs4504 |
| TIMP3 | rs135029 |
| TIMP3 | rs5754312 |
| TIMP3 | rs2267183 |
| TIMP3 | rs1427378 |
| TIMP3 | rs1427376 |
| TIMP3 | rs9862 |
| TIMP3 | rs137485 |
| TIMP3 | rs137487 |
| TIMP3 | rs5749529 |
| TIMP3 | rs137489 |
| TIMP3 | rs2040435 |
| LTA | rs2844482 |
| LTA | rs2229094 |
| LTA | rs1799964 |
| HSPA1L | rs2075800 |
| HSPA1L | rs2227956 |
| HSPA1A | rs1043618 |
| HSPA1A | rs2763979 |
| C6orf48 | rs2471980 |
| KL | rs398655 |
| KL | rs495392 |
| KL | rs526906 |
| KL | rs9526983 |
| KL | rs9536239 |
| KL | rs577912 |
| KL | rs1207362 |
| KL | rs685417 |
| KL | rs1334928 |
| KL | rs1888057 |
| KL | rs657049 |
| KL | rs9527025 |
| KL | rs2149860 |
| KL | rs522796 |
| KL | rs648202 |
| KL | rs643780 |
| KL | rs659117 |
| KL | rs582524 |
| IL10RB | rs2284552 |
| IL10RB | rs2834168 |
| IL10RB | rs2243498 |
| IL10RB | rs2834172 |
| IL10RB | rs765429 |
| IL10RB | rs999261 |
| IL10RB | rs999259 |
| IL10RB | rs1058867 |
| IL10RB | rs6517158 |
| IL10RB | rs2834175 |
| NFKBIA | rs696 |
| NFKBIA | rs3138045 |
| IL2RB | rs2743827 |
| IL2RB | rs228937 |
| IL2RB | rs3218339 |
| IL2RB | rs3218329 |
| IL2RB | rs84458 |
| IL2RB | rs84460 |
| IL2RB | rs228945 |
| IL2RB | rs228947 |
| IL2RB | rs2072861 |
| IL2RB | rs3218315 |
| IL2RB | rs3218312 |
| IL2RB | rs228954 |
| IL2RB | rs3218295 |
| IL2RB | rs3218292 |
| IL2RB | rs228957 |
| IL2RB | rs2281094 |
| IL2RB | rs228968 |
| IL2RB | rs1003694 |
| IL2RB | rs2235330 |
| IL2RB | rs3218264 |
| IL2RB | rs228975 |
| PGEA1 | rs6001188 |
| TOMM22 | rs5757231 |
| TOMM22 | rs6001193 |
| CYP2D6 | rs5758589 |
| CRHR1 | rs17689966 |
| IMP5 | rs878887 |
| TREM1 | rs16894387 |
| TREM1 | rs2234243 |
| TREM1 | rs1817537 |
| TREM1 | rs3804277 |
| TREM1 | rs4711668 |
| TREM1 | rs6910730 |
| TREM1 | rs6940092 |
| TREM1 | rs6939973 |
| TREM1 | rs3827632 |
| TREM1 | rs1385105 |
| EDN2 | rs12069358 |
| EDN2 | rs1407550 |
| EDN2 | rs6690839 |
| EDN2 | rs11572340 |
| EDN2 | rs3754287 |
| EDN2 | rs1077218 |
| EDN2 | rs883304 |
| EDN2 | rs12718439 |
| EDN2 | rs4660541 |
| AP3M2 | rs4581040 |
| AP3M2 | rs4471024 |
| PLAT | rs2020922 |
| PLAT | rs879293 |
| PLAT | rs2299609 |
| PLAT | rs7837156 |
| PLAT | rs2020919 |
| PTCRA | rs16896153 |
| PTCRA | rs9471960 |
| PTCRA | rs6901007 |
| TNRC5 | rs2234185 |
| CBS | rs706208 |
| CBS | rs1051319 |
| CBS | rs12329764 |
| CBS | rs234705 |
| CBS | rs234709 |
| CBS | rs234715 |
| CBS | rs11701048 |
| CBS | rs1788484 |
| VEGF | rs699947 |
| VEGF | rs833068 |
| VEGF | rs833069 |
| VEGF | rs3025010 |
| VEGF | rs3025033 |
| VEGF | rs998584 |
| VEGF | rs6900017 |
| MMP9 | rs4810482 |
| MMP9 | rs8113877 |
| MMP9 | rs6104420 |
| NFKBIB | rs11575002 |
| NFKBIB | rs2053071 |
| NFKBIB | rs2241704 |
| NFKBIB | rs2241705 |
| NFKBIB | rs3136641 |
| NFKBIB | rs3136646 |
| SLC35B2 | rs1875324 |
| SLC35B2 | rs2282151 |
| NFKBIE | rs730775 |
| NFKBIE | rs483536 |
| HILS1 | rs2586482 |
| HILS1 | rs2586485 |
| COL1A1 | rs1061237 |
| COL1A1 | rs2277632 |
| COL1A1 | rs2586488 |
| COL1A1 | rs2075559 |
| COL1A1 | rs2857396 |
| COL1A1 | rs2696247 |
| COL1A1 | rs2269336 |
| COL1A1 | rs1107946 |
| IGFBP3 | rs2471551 |
| MGC20255 | rs10417924 |
| MGC4093 | rs1982072 |
| F2 | rs2070852 |
| F2 | rs3136485 |
| IGSF4C | rs4803648 |
| IGSF4C | rs4802189 |
| PLAUR | rs4251938 |
| PLAUR | rs2302524 |
| PLAUR | rs4251864 |
| PLAUR | rs2239372 |
| PLAUR | rs2283628 |
| PLAUR | rs397374 |
| PLAUR | rs4251854 |
| PLAUR | rs4251831 |
| PLAUR | rs344787 |
| PLAUR | rs2286960 |
| PLAUR | rs344781 |
| PLAUR | rs344779 |
| CYP19A1 | rs4775932 |
| CARD15 | rs4785224 |
| CYP19A1 | rs4275794 |
| CYP19A1 | rs2899470 |
| CARD15 | rs17312836 |
| CARD15 | rs2066843 |
| CYP19A1 | rs2899472 |
| CYP19A1 | rs4775935 |
| CARD15 | rs751271 |
| CARD15 | rs5743289 |
| CARD15 | rs5743291 |
| CYP19A1 | rs700518 |
| CYP19A1 | rs17703883 |
| CYP19A1 | rs10519295 |
| CYP19A1 | rs727479 |
| CYP19A1 | rs10459592 |
| CARD15 | rs8056611 |
| CYP19A1 | rs12591359 |
| CYP19A1 | rs767199 |
| CYP19A1 | rs12911554 |
| CYP19A1 | rs7172156 |
| CYP19A1 | rs11856927 |
| CYP19A1 | rs4545755 |
| CYP19A1 | rs1054984 |
| CYP19A1 | rs4614671 |
| CYP19A1 | rs12050767 |
| CYP19A1 | rs749292 |
| CYP19A1 | rs2305707 |
| CYP19A1 | rs16953058 |
| CYP19A1 | rs936306 |
| CYP19A1 | rs936307 |
| CYP19A1 | rs2470176 |
| CYP19A1 | rs17523880 |
| CYP19A1 | rs2470152 |
| CYP19A1 | rs17523922 |
| CYP19A1 | rs3751592 |
| CYP19A1 | rs3751591 |
| CYP19A1 | rs2470150 |
| CYP19A1 | rs1902584 |
| CYP19A1 | rs1004982 |
| CYP19A1 | rs1902585 |
| CYP19A1 | rs10163138 |
| CYP19A1 | rs7174997 |
| CYP19A1 | rs8025191 |
| CYP19A1 | rs1961177 |
| PTGER2 | rs1390376 |
| PTGER2 | rs1254600 |
| PTGER2 | rs1254593 |
| PTGER2 | rs708498 |
| PTGER2 | rs12147805 |
| PTGER2 | rs708506 |
| PTK9L | rs352143 |
| MMP2 | rs243866 |
| MMP2 | rs1477017 |
| MMP2 | rs865094 |
| MMP2 | rs11646643 |
| MMP2 | rs1053605 |
| MMP2 | rs866770 |
| MMP2 | rs2241145 |
| MMP2 | rs243845 |
| MMP2 | rs243842 |
| MMP2 | rs183112 |
| MMP2 | rs1992116 |
| MMP2 | rs11639960 |
| MMP2 | rs243836 |
| MMP2 | rs243834 |
| MMP2 | rs11541998 |
| MMP2 | rs243832 |
| MMP2 | rs243831 |
| MMP2 | rs9922534 |
| MMP2 | rs2241148 |
| MMP2 | rs8054459 |
| MMP2 | rs2192853 |
| MBL2 | rs16933062 |
| MBL2 | rs12771266 |
| MBL2 | rs2506 |
| MBL2 | rs10082466 |
| MBL2 | rs930507 |
| MBL2 | rs1838065 |
| MBL2 | rs1838066 |
| MBL2 | rs10824793 |
| MBL2 | rs11003123 |
| MBL2 | rs10824796 |
| MBL2 | rs11003129 |
| ACE | rs4295 |
| ACE | rs4305 |
| ACE | rs4311 |
| ACE | rs4362 |
| ACE | rs4461142 |
| ACE | rs4267385 |
| ACE | rs4459610 |
| KCNH6 | rs12451328 |
| SCGB1A1 | rs10897270 |
| SCGB1A1 | rs3741240 |
| SCGB1A1 | rs17145874 |
| MTHFD1 | rs1956545 |
| MTHFD1 | rs3783731 |
| MTHFD1 | rs1950902 |
| MTHFD1 | rs17751556 |
| MTHFD1 | rs2295640 |
| MTHFD1 | rs17824591 |
| MTHFD1 | rs1885031 |
| MTHFD1 | rs8016556 |
| MTHFD1 | rs2236225 |
| MTHFD1 | rs3818239 |
| MTHFD1 | rs11849530 |
| MTHFD1 | rs1256146 |
| AKAP5 | rs745686 |
| IFNG | rs10878763 |
| IFNG | rs2069727 |
| IFNG | rs2069718 |
| IFNG | rs2069716 |
| IFNG | rs2069705 |
| GSTP1 | rs947895 |
| CRH | rs10105164 |
| CRH | rs6996265 |
| CRH | rs3176921 |
| CRH | rs6472257 |
| CRH | rs7839698 |
| CRH | rs10098823 |
| PTGER3 | rs959 |
| PTGER3 | rs1327460 |
| PTGER3 | rs6656853 |
| PTGER3 | rs7530345 |
| PTGER3 | rs6685546 |
| PTGER3 | rs17131465 |
| PTGER3 | rs12119442 |
| PTGER3 | rs1409985 |
| PTGER3 | rs1327449 |
| PTGER3 | rs4649932 |
| PTGER3 | rs1409981 |
| PTGER3 | rs4147115 |
| PTGER3 | rs1409165 |
| PTGER3 | rs4650094 |
| PTGER3 | rs875727 |
| PTGER3 | rs17541722 |
| PTGER3 | rs1327466 |
| PTGER3 | rs1887404 |
| PTGER3 | rs17542063 |
| PTGER3 | rs6424410 |
| PTGER3 | rs602383 |
| PTGER3 | rs578096 |
| PTGER3 | rs6670616 |
| PTGER3 | rs2421735 |
| PTGER3 | rs977214 |
| PTGER3 | rs6665776 |
| PTGER3 | rs594454 |
| PTGER3 | rs2300161 |
| PTGER3 | rs5697 |
| PTGER3 | rs2072947 |
| PTGER3 | rs481940 |
| PTGER3 | rs3819783 |
| PTGER3 | rs3819790 |
| PTGER3 | rs726764 |
| PTGER3 | rs1409164 |
| PTGER3 | rs2256385 |
| PTGER3 | rs2300164 |
| PTGER3 | rs2050066 |
| PTGER3 | rs6424414 |
| PTGER3 | rs2300167 |
| PTGER3 | rs6678886 |
| PTGER3 | rs10789314 |
| PTGER3 | rs5693 |
| PTGER3 | rs5680 |
| PTGER3 | rs5673 |
| PTGER3 | rs11209736 |
| PTGER3 | rs8179390 |
| PTGER3 | rs2817864 |
| PTGER3 | rs3000466 |
| CYP1A1 | rs2470893 |
| IL8 | rs4694178 |
| IL8 | rs4694637 |
| SERPINH1 | rs646474 |
| SERPINH1 | rs649257 |
| C10orf55 | rs2227551 |
| PLAU | rs2227562 |
| PLAU | rs2227564 |
| PLAU | rs34930250 |
| PLAU | rs3805118 |
| PLAU | rs2461863 |
| F2R | rs2227744 |
| F2R | rs27593 |
| F2R | rs37249 |
| F2R | rs153311 |
| F2R | rs250738 |
| F2R | rs11954573 |
| F2R | rs250731 |
| F2RL1 | rs6453251 |
| F2RL1 | rs639342 |
| F2RL1 | rs2242991 |
| F2RL1 | rs2243004 |
| F2RL1 | rs2243010 |
| F2RL1 | rs34308580 |
| F2RL1 | rs631465 |
| F2RL1 | rs2243083 |
| F2RL1 | rs2243066 |
| F2RL1 | rs6453253 |
| CRHBP | rs32897 |
| CRHBP | rs6453267 |
| CRHBP | rs10055255 |
| CRHBP | rs1875999 |
| CRHBP | rs10514082 |
| PTGFR | rs3766354 |
| PTGFR | rs1555541 |
| PTGFR | rs1322934 |
| PTGFR | rs6701594 |
| PTGFR | rs12725125 |
| PTGFR | rs3766345 |
| PTGFR | rs668005 |
| PTGFR | rs622346 |
| PTGFR | rs3766333 |
| PTGFR | rs12074883 |
| DHFR | rs12517451 |
| DHFR | rs1650723 |
| DHFR | rs1677693 |
| DHFR | rs10072026 |
| DHFR | rs1650697 |
| DHFR | rs380691 |
| TSHR | rs8009058 |
| TSHR | rs2268451 |
| TSHR | rs12892567 |
| TSHR | rs179247 |
| TSHR | rs179259 |
| TSHR | rs179260 |
| TSHR | rs179261 |
| TSHR | rs2110696 |
| TSHR | rs1035145 |
| TSHR | rs3783950 |
| TSHR | rs3783948 |
| TSHR | rs7143071 |
| TSHR | rs17111361 |
| TSHR | rs6574616 |
| TSHR | rs724169 |
| TSHR | rs2300520 |
| TSHR | rs4903964 |
| TSHR | rs917986 |
| TSHR | rs17545310 |
| TSHR | rs3783943 |
| TSHR | rs2300521 |
| TSHR | rs12881268 |
| TSHR | rs2268466 |
| TSHR | rs4903967 |
| TSHR | rs7161100 |
| TSHR | rs2300525 |
| TSHR | rs2110697 |
| TSHR | rs1005292 |
| TSHR | rs17111394 |
| TSHR | rs2268474 |
| TSHR | rs2300528 |
| TSHR | rs2284735 |
| TSHR | rs2075173 |
| TSHR | rs17111431 |
| TSHR | rs10129380 |
| TSHR | rs7150670 |
| TSHR | rs2268475 |
| TSHR | rs11159491 |
| TSHR | rs2024426 |
| TSHR | rs11845715 |
| TSHR | rs12885526 |
| TSHR | rs8017455 |
| TSHR | rs917984 |
| TSHR | rs7158881 |
| TSHR | rs6574629 |
| TSHR | rs2268476 |
| TSHR | rs3783938 |
| TSHR | rs17111530 |
| TSHR | rs930099 |
| TSHR | rs7157900 |
| TSHR | rs1957547 |
| TSHR | rs2300540 |
| TSHR | rs1991517 |
| TSHR | rs2268477 |
| TSHR | rs7144481 |
| TSHR | rs17630128 |
| TSHR | rs2288493 |
| TSHR | rs12883801 |
| FAS | rs983751 |
| FAS | rs4934434 |
| FAS | rs3758483 |
| FAS | rs6586165 |
| FAS | rs1571011 |
| FAS | rs9658727 |
| FAS | rs7901656 |
| FAS | rs2031611 |
| FAS | rs9658761 |
| FAS | rs982764 |
| FAS | rs2234978 |
| FAS | rs1051070 |
| FAS | rs7915235 |
| COL1A2 | rs11765563 |
| COL1A2 | rs388625 |
| COL1A2 | rs1800222 |
| COL1A2 | rs411717 |
| COL1A2 | rs420257 |
| COL1A2 | rs760043 |
| COL1A2 | rs406226 |
| COL1A2 | rs3763466 |
| COL1A2 | rs17166249 |
| COL1A2 | rs389328 |
| COL1A2 | rs42521 |
| COL1A2 | rs42523 |
| COL1A2 | rs42524 |
| COL1A2 | rs2621213 |
| COL1A2 | rs2521205 |
| COL1A2 | rs7781954 |
| COL1A2 | rs42527 |
| COL1A2 | rs369982 |
| COL1A2 | rs42528 |
| COL1A2 | rs4266 |
| COL1A2 | rs2472 |
| COL1A2 | rs42531 |
| COL1A2 | rs441051 |
| COL1A2 | rs400218 |
| COL1A2 | rs7804898 |
| COL1A2 | rs10046552 |
| COL1A2 | rs6465412 |
| COL1A2 | rs12668754 |
| COL1A2 | rs11764718 |
| COL1A2 | rs1062394 |
| COL1A2 | rs11982782 |
| COL1A2 | rs13234022 |
| PON1 | rs854547 |
| PON1 | rs8491 |
| PON1 | rs854548 |
| PON1 | rs3735590 |
| PON1 | rs854551 |
| PON1 | rs854552 |
| PON1 | rs854555 |
| PON1 | rs3917550 |
| PON1 | rs2269829 |
| PON1 | rs3917542 |
| PON1 | rs662 |
| PON1 | rs3917538 |
| F3 | rs762485 |
| F3 | rs762484 |
| F3 | rs696619 |
| F3 | rs28672143 |
| PON1 | rs854560 |
| PON1 | rs2272365 |
| PON1 | rs3917490 |
| PON1 | rs2049649 |
| PON1 | rs2299260 |
| PON1 | rs2299261 |
| PON1 | rs854568 |
| PON1 | rs2299262 |
| PON1 | rs2237583 |
| PON1 | rs757158 |
| PON2 | rs11977702 |
| PON2 | rs9641164 |
| PON2 | rs987539 |
| PON2 | rs2286232 |
| PON2 | rs2299266 |
| PON2 | rs2237585 |
| PON2 | rs2286233 |
| PON2 | rs11981433 |
| PON2 | rs7802018 |
| PON2 | rs2299267 |
| PON2 | rs730365 |
| PON2 | rs43037 |
| PON2 | rs6978425 |
| PGR | rs11224561 |
| PGR | rs471767 |
| PGR | rs563656 |
| PGR | rs504372 |
| PGR | rs578029 |
| PGR | rs635984 |
| PGR | rs11224575 |
| PGR | rs492457 |
| PGR | rs518382 |
| PGR | rs553272 |
| PGR | rs660149 |
| ADH1B | rs12507573 |
| ADH1B | rs1042026 |
| ADH1B | rs17033 |
| ADH1B | rs13133908 |
| PGR | rs653752 |
| ADH1B | rs1789882 |
| ADH1B | rs1693457 |
| ADH1B | rs4147536 |
| ADH1B | rs1353621 |
| ADH1B | rs1159918 |
| ADH1B | rs1229982 |
| PGR | rs538915 |
| PGR | rs503362 |
| PGR | rs542384 |
| ADH1C | rs1229980 |
| PGR | rs555572 |
| PGR | rs11224589 |
| ADH1C | rs1614972 |
| PGR | rs619487 |
| ADH1C | rs904096 |
| ADH1C | rs3762896 |
| PGR | rs537681 |
| ADH1C | rs17586163 |
| PGR | rs518162 |
| PGR | rs507141 |
| SERPINE1 | rs6950982 |
| SERPINE1 | rs2227631 |
| SERPINE1 | rs2227667 |
| SERPINE1 | rs2227672 |
| SERPINE1 | rs2070682 |
| SERPINE1 | rs1050813 |
| SERPINE1 | rs11560324 |
| IGF1 | rs1520220 |
| IL1R2 | rs11884283 |
| IL1R2 | rs6543105 |
| IL1R2 | rs12467316 |
| IL1R2 | rs4141134 |
| IL1R2 | rs4851520 |
| IL1R2 | rs4851522 |
| IL1R2 | rs4321386 |
| IL1R2 | rs1108338 |
| IL1R2 | rs7561191 |
| IL1R2 | rs4851526 |
| IL1R2 | rs4851527 |
| IL1R2 | rs2302589 |
| IL1R2 | rs2160140 |
| IL1R2 | rs3218883 |
| IL1R2 | rs3218927 |
| IL1R2 | rs2072474 |
| IL1R2 | rs3218979 |
| IL1R2 | rs733498 |
| IL1R2 | rs7589525 |
| IL1R2 | rs4851531 |
| MMP8 | rs1276284 |
| MMP8 | rs2508383 |
| MMP8 | rs1939020 |
| MMP8 | rs17099443 |
| MMP8 | rs1940475 |
| MMP8 | rs11225394 |
| MMP8 | rs6590985 |
| MMP8 | rs10895354 |
| IL1R1 | rs3917225 |
| IL1R1 | rs949963 |
| IL1R1 | rs3771202 |
| IL1R1 | rs2287047 |
| IL1R1 | rs3917254 |
| IL1R1 | rs3917292 |
| IL1R1 | rs3917296 |
| IL1R1 | rs951193 |
| IL1R1 | rs3171845 |
| IL1R1 | rs2110726 |
| IL1R1 | rs3732131 |
| MMP1 | rs1939008 |
| IL1R1 | rs3917332 |
| MMP1 | rs17293823 |
| MMP1 | rs7945189 |
| MMP1 | rs2071230 |
| MMP1 | rs470747 |
| MMP1 | rs7125062 |
| MMP1 | rs5031036 |
| MMP1 | rs996999 |
| MMP1 | rs470358 |
| MMP1 | rs1155764 |
| MMP1 | rs484915 |
| MMP3 | rs569444 |
| MMP3 | rs650108 |
| MMP3 | rs520540 |
| MMP3 | rs522616 |
| MMP3 | rs645419 |
| NFKB1 | rs980455 |
| NFKB1 | rs3774933 |
| NFKB1 | rs1599961 |
| NFKB1 | rs1585213 |
| NFKB1 | rs230528 |
| NFKB1 | rs13117745 |
| NFKB1 | rs1801 |
| NFKB1 | rs4648058 |
| NFKB1 | rs3755867 |
| NFKB1 | rs4648090 |
| NFKB1 | rs3817685 |
| NFKB1 | rs4648135 |
| NFKB1 | rs4648141 |
| NFKB1 | rs1609798 |
| NFKB1 | rs7674640 |
| NFKB1 | rs997476 |
| NFKB1 | rs10489113 |
| NFKB2 | rs11574845 |
| NFKB2 | rs7897947 |
| NFKB2 | rs1056890 |
| IL18 | rs543810 |
| IL18 | rs5744280 |
| IL18 | rs360722 |
| IL18 | rs4937113 |
| IL18 | rs2043055 |
| TEX12 | rs1946519 |
| TEX12 | rs5744222 |
| TEX12 | rs1293344 |
| F7 | rs555212 |
| F7 | rs488703 |
| F7 | rs6046 |
| F7 | rs3093261 |
| F7 | rs3211719 |
| F10 | rs3212998 |
| F10 | rs474810 |
| F10 | rs3211744 |
| F10 | rs547138 |
| F10 | rs3211764 |
| F10 | rs2026160 |
| F10 | rs3211770 |
| F10 | rs9549675 |
| F10 | rs559054 |
| F10 | rs5960 |
| IL1A | rs17561 |
| IL1A | rs2856838 |
| IL1A | rs1878321 |
| IL1B | rs2853550 |
| IL1B | rs1143643 |
| IL1B | rs1143634 |
| IL1B | rs1143630 |
| IL1B | rs1143627 |
| IL1B | rs1143623 |
| IL1B | rs4848306 |
| IL1RN | rs17042917 |
| IL1RN | rs315920 |
| IL1RN | rs4251961 |
| IL1RN | rs2637988 |
| IL1RN | rs928940 |
| IL1RN | rs3213448 |
| IL1RN | rs1794066 |
| IL1RN | rs380092 |
| IL1RN | rs579543 |
| IL1RN | rs315951 |
| IL1RN | rs315949 |
| IL1RN | rs315946 |
| IL1RN | rs315943 |
| IL1RN | rs315942 |
| PTPN22 | rs2476601 |
| PAFAH1B2 | rs4938347 |
| PAFAH1B2 | rs3736120 |
| IL10RA | rs4936414 |
| IL10RA | rs2512143 |
| IL10RA | rs4252254 |
| IL10RA | rs4252270 |
| IL10RA | rs2229113 |
| IL10RA | rs9610 |
| IL10RA | rs2508445 |
| IL10RA | rs947889 |
| IL10RA | rs4938467 |
| IL10RA | rs11216666 |
| IL10RA | rs17121510 |
| TLR4 | rs10759930 |
| TLR4 | rs2770150 |
| TLR4 | rs10759932 |
| TLR4 | rs1927911 |
| TLR4 | rs2149356 |
| TLR4 | rs11536889 |
| TLR4 | rs1927906 |
| TLR4 | rs11536898 |
| TLR4 | rs1554973 |
| TLR4 | rs7856729 |
| IL2 | rs10027390 |
| IL2 | rs2069772 |
| IL2 | rs2069771 |
| IL2 | rs2069779 |
| IL2 | rs2069778 |
| IL2 | rs2069762 |
| IL2 | rs4833248 |
| PTGS1 | rs1330344 |
| PTGS1 | rs1213266 |
| PTGS1 | rs10306135 |
| PTGS1 | rs7866582 |
| PTGS1 | rs10306150 |
| PTGS1 | rs4273915 |
| PTGS1 | rs4240474 |
| PTGS1 | rs3842798 |
| PTGS1 | rs9299282 |
| PTGS1 | rs12238505 |
| PTGS1 | rs10306202 |
| PGRMC2 | rs11726595 |
| PGRMC2 | rs2036687 |
| PGRMC2 | rs4975220 |
| PGRMC2 | rs4975180 |
| PGRMC2 | rs3733260 |
| PTGES | rs11999368 |
| PTGES | rs4636306 |
| PTGES | rs2302821 |
| PTGES | rs10739757 |
| PTGES | rs2241270 |
| PTGES | rs10988496 |
| IL5 | rs743562 |
| IL5 | rs739719 |
| IL5 | rs739718 |
| IL13 | rs3091307 |
| IL13 | rs1295686 |
| IL13 | rs848 |
| IL13 | rs1295683 |
| IL13 | rs2243204 |
| IL4 | rs2243248 |
| IL4 | rs2070874 |
| IL4 | rs2227284 |
| IL4 | rs2243263 |
| IL4 | rs2243268 |
| IL4 | rs2243274 |
| IL4 | rs2243290 |
| HSPA4 | rs4574536 |
| HSPA4 | rs4705990 |
| HSPA4 | rs10075878 |
| COL5A1 | rs12002679 |
| COL5A1 | rs3124291 |
| COL5A1 | rs3128597 |
| COL5A1 | rs3124311 |
| COL5A1 | rs4842151 |
| COL5A1 | rs4842157 |
| COL5A1 | rs4842161 |
| COL5A1 | rs3124932 |
| COL5A1 | rs12005720 |
| COL5A1 | rs3128621 |
| COL5A1 | rs4842167 |
| COL5A1 | rs3811161 |
| COL5A1 | rs11999194 |
| COL5A1 | rs3811153 |
| COL5A1 | rs3811152 |
| COL5A1 | rs3811151 |
| COL5A1 | rs10776908 |
| COL5A1 | rs10745387 |
| COL5A1 | rs4842172 |
| COL5A1 | rs4841937 |
| COL5A1 | rs11103543 |
| COL5A1 | rs13946 |
| SLC23A1 | rs6596471 |
| SLC23A1 | rs6596473 |
| SLC23A1 | rs11950646 |
| TRAF2 | rs2784075 |
| TRAF2 | rs908831 |
| CD14 | rs2569190 |
| CD14 | rs2569193 |
| NR3C1 | rs17287758 |
| NR3C1 | rs10482682 |
| NR3C1 | rs4986593 |
| NR3C1 | rs33388 |
| NR3C1 | rs17100236 |
| NR3C1 | rs2918417 |
| NR3C1 | rs2963155 |
| NR3C1 | rs9324918 |
| NR3C1 | rs9324921 |
| NR3C1 | rs4634384 |
| NR3C1 | rs9324924 |
| NR3C1 | rs7701443 |
| NR3C1 | rs4244032 |
| NR3C1 | rs4607376 |
| NR3C1 | rs13182800 |
| IL15 | rs12508866 |
| NR3C1 | rs4912911 |
| NR3C1 | rs12656106 |
| NR3C1 | rs12655166 |
| IL15 | rs1519551 |
| IL15 | rs17461269 |
| IL15 | rs1519552 |
| IL15 | rs7698675 |
| IL15 | rs13117878 |
| IL15 | rs12498901 |
| IL15 | rs6850492 |
| IL15 | rs1907949 |
| IL15 | rs17007610 |
| IL15 | rs6537061 |
| ADRB2 | rs1432622 |
| ADRB2 | rs12654778 |
| ADRB2 | rs1042713 |
| ADRB2 | rs4705271 |
| NOS3 | rs12703107 |
| NOS3 | rs1800783 |
| NOS3 | rs1799983 |
| NOS3 | rs3918227 |
| NOS3 | rs743507 |
| ATG9B | rs2373929 |
| IL6R | rs952146 |
| IL6R | rs1386821 |
| IL6R | rs4075015 |
| IL6R | rs4845618 |
| IL6R | rs6687726 |
| IL6R | rs7549338 |
| IL6R | rs4553185 |
| IL6R | rs4845622 |
| IL6R | rs4845623 |
| IL6R | rs4537545 |
| IL6R | rs4845625 |
| IL6R | rs4845374 |
| IL6R | rs11265618 |
| IL6R | rs10752641 |
| IL6R | rs4329505 |
| IL6R | rs2229238 |
| IL6R | rs4072391 |
| SHE | rs7526293 |
| TLR2 | rs1898830 |
| TLR2 | rs4696483 |
| TLR2 | rs7656411 |
| TLR2 | rs1337 |
| CRP | rs3093066 |
| CRP | rs1800947 |
| CRP | rs1417938 |
| HSPA6 | rs9427401 |
| HSPA6 | rs12129787 |
| HSPA6 | rs4657054 |
| HSPA6 | rs404508 |
| HSPA6 | rs2099684 |
| HSD17B7 | rs1780019 |
| PLG | rs783144 |
| HSD17B7 | rs4656381 |
| PLG | rs2314852 |
| HSD17B7 | rs1039874 |
| PLG | rs1950562 |
| PLG | rs9458011 |
| HSD17B7 | rs2805053 |
| PLG | rs1652508 |
| PLG | rs4252092 |
| HSD17B7 | rs2803865 |
| PLG | rs9295131 |
| PLG | rs783147 |
| PLG | rs4252125 |
| PLG | rs813641 |
| PLG | rs4252159 |
| PLG | rs4252166 |
| PLG | rs783176 |
| PLG | rs11060 |
| PLG | rs9458023 |
| PLG | rs783166 |
| F5 | rs2187952 |
| F5 | rs2420369 |
| F5 | rs2213865 |
| F5 | rs3766103 |
| F5 | rs12131397 |
| F5 | rs9332618 |
| F5 | rs6427197 |
| F5 | rs4656687 |
| F5 | rs1557572 |
| F5 | rs6035 |
| F5 | rs12120605 |
| F5 | rs6427198 |
| F5 | rs1894697 |
| F5 | rs9332575 |
| F5 | rs6022 |
| F5 | rs9287095 |
| F5 | rs2298908 |
| F5 | rs6019 |
| F5 | rs12755775 |
| F5 | rs10489185 |
| SELP | rs3917854 |
| FASLG | rs2859242 |
| FASLG | rs2639614 |
| FASLG | rs6700734 |
| FASLG | rs17370527 |
| FASLG | rs5030772 |
| FASLG | rs12041613 |
| PTGS2 | rs2066826 |
| PTGS2 | rs2745557 |
| PTGS2 | rs689466 |
| PTGS2 | rs12042763 |
| PTGS2 | rs10911905 |
| PTGS2 | rs2179555 |
| PLA2G4A | rs4651330 |
| PLA2G4A | rs2076075 |
| PLA2G4A | rs6696406 |
| PLA2G4A | rs12404877 |
| PLA2G4A | rs12720497 |
| PLA2G4A | rs6685652 |
| PLA2G4A | rs2223307 |
| PLA2G4A | rs17591814 |
| PLA2G4A | rs2223309 |
| PLA2G4A | rs1980444 |
| PLA2G4A | rs2049963 |
| PLA2G4A | rs10911946 |
| PLA2G4A | rs12749354 |
| PLA2G4A | rs7540602 |
| PLA2G4A | rs6695515 |
| PLA2G4A | rs726706 |
| PLA2G4A | rs6656909 |
| PLA2G4A | rs1569479 |
| PLA2G4A | rs6683515 |
| PLA2G4A | rs12726519 |
| PLA2G4A | rs6683416 |
| PLA2G4A | rs11587539 |
| PLA2G4A | rs7555140 |
| PLA2G4A | rs932476 |
| PLA2G4A | rs10157410 |
| PLA2G4A | rs7545121 |
| PLA2G4A | rs4402086 |
| PLA2G4A | rs7526089 |
| PLA2G4A | rs761517 |
| TLR3 | rs4862632 |
| TLR3 | rs5743303 |
| TLR3 | rs5743305 |
| TLR3 | rs11721827 |
| TLR3 | rs5743312 |
| TLR3 | rs7668666 |
| TLR3 | rs3775292 |
| TLR3 | rs3775291 |
| TLR3 | rs4862633 |
| TFPI | rs12693471 |
| TFPI | rs8176541 |
| TFPI | rs7586970 |
| TFPI | rs3213739 |
| TFPI | rs8176508 |
| TFPI | rs2041778 |
| TFPI | rs2192824 |
| TFPI | rs3755248 |
| TFPI | rs12613071 |
| TFPI | rs16829086 |
| TFPI | rs7573488 |
| TFPI | rs7594359 |
| TFPI | rs10179730 |
| TFPI | rs6434222 |
| TFPI | rs10187622 |
| COL3A1 | rs2138533 |
| COL3A1 | rs1878201 |
| COL3A1 | rs1516454 |
| COL3A1 | rs1914037 |
| COL3A1 | rs17358825 |
| COL3A1 | rs16830973 |
| COL3A1 | rs3736487 |
| COL3A1 | rs3134656 |
| COL3A1 | rs12693525 |
| COL3A1 | rs7579903 |
| COL3A1 | rs2271682 |
| COL3A1 | rs10204508 |
| COL3A1 | rs2203602 |
| COL3A1 | rs3134646 |
| COL3A1 | rs4667256 |
| COL3A1 | rs4667258 |
| COL5A2 | rs6760780 |
| COL5A2 | rs3923384 |
| COL5A2 | rs6434317 |
| COL5A2 | rs6434322 |
| COL5A2 | rs10165260 |
| COL5A2 | rs7420331 |
| COL5A2 | rs13024858 |
| COL5A2 | rs6752781 |
| COL5A2 | rs1515864 |
| COL5A2 | rs9288163 |
| COL5A2 | rs1399991 |
| COL5A2 | rs10497699 |
| COL5A2 | rs12611950 |
| COL5A2 | rs11691604 |
| IL1RAP | rs9817203 |
| IL1RAP | rs7628333 |
| IL1RAP | rs3821744 |
| IL1RAP | rs9290936 |
| IL1RAP | rs9849030 |
| IL1RAP | rs2059020 |
| IL1RAP | rs7626071 |
| IL1RAP | rs3773990 |
| IL1RAP | rs2193880 |
| IL1RAP | rs3773989 |
| IL1RAP | rs1988743 |
| IL1RAP | rs4686554 |
| IL1RAP | rs16865597 |
| IL1RAP | rs2885370 |
| IL1RAP | rs3773983 |
| IL1RAP | rs3773982 |
| IL1RAP | rs3773981 |
| IL1RAP | rs9883249 |
| IL1RAP | rs9845825 |
| IL1RAP | rs2241343 |
| IL1RAP | rs9877268 |
| IL1RAP | rs3773977 |
| IL1RAP | rs3773976 |
| IL1RAP | rs4687151 |
| IL1RAP | rs11929157 |
| IL1RAP | rs10937439 |
| IL1RAP | rs10937442 |
| IL1RAP | rs1035347 |
| IL1RAP | rs12053868 |
| IL1RAP | rs6444435 |
| IL1RAP | rs4687154 |
| IL1RAP | rs10513854 |
| IL1RAP | rs7628250 |
| IL1RAP | rs3773958 |
| IL1RAP | rs3773953 |
| IL1RAP | rs1469007 |
| IL1RAP | rs9875362 |
| IL1RAP | rs6781037 |
| IL1RAP | rs6765375 |
| IL1RAP | rs759783 |
| IL1RAP | rs4140711 |
| IL1RAP | rs9290939 |
| IL1RAP | rs1015704 |
| IL1RAP | rs1015705 |
| IL1RAP | rs4687163 |
| IL1RAP | rs929729 |
| IL1RAP | rs1024941 |
| IL1RAP | rs1024946 |
| IL1RAP | rs1024949 |
| IL1RAP | rs7626795 |
| IL1RAP | rs4624606 |
| IL1RAP | rs9847868 |
| IL1RAP | rs9821122 |
| IL1RAP | rs4320092 |
| IL1RAP | rs9831803 |
| IL1RAP | rs7650510 |
| IL1RAP | rs11915384 |
| CTLA4 | rs231770 |
| CTLA4 | rs16840252 |
| CTLA4 | rs11571317 |
| CTLA4 | rs5742909 |
| CTLA4 | rs231777 |
| CTLA4 | rs231779 |
| CTLA4 | rs3087243 |
| CTLA4 | rs231726 |
| CTLA4 | rs231727 |
| IL10 | rs3024498 |
| IL10 | rs3024496 |
| IL10 | rs1800872 |
| IL10 | rs1800896 |
| G0S2 | rs17389016 |
| HSD11B1 | rs2235543 |
| HSD11B1 | rs4844880 |
| HSD11B1 | rs846910 |
| HSD11B1 | rs3753519 |
| HSD11B1 | rs6672256 |
| HSD11B1 | rs9430012 |
| HSD11B1 | rs932335 |
| IL8RA | rs1008563 |
| IL8RA | rs1008562 |
| IL8RA | rs2854386 |
| EPHX1 | rs2854450 |
| EPHX1 | rs3753658 |
| EPHX1 | rs1877724 |
| EPHX1 | rs2671272 |
| EPHX1 | rs2260863 |
| EPHX1 | rs2740168 |
| EPHX1 | rs2740170 |
| EPHX1 | rs1051741 |
| EPHX1 | rs360063 |
| UGT1A3 | rs3755319 |
| UGT1A1 | rs6742078 |
| UGT1A1 | rs4148324 |
| UGT1A1 | rs929596 |
| UGT1A1 | rs2302538 |
| UGT1A1 | rs11888492 |
| UGT1A1 | rs10929303 |
| UGT1A1 | rs8330 |
| UGT1A1 | rs4148329 |
| UGT1A1 | rs6717546 |
| UGT1A1 | rs1500482 |
| UGT1A1 | rs4663972 |
| UGT1A1 | rs7586006 |
| MTR | rs12759827 |
| MTR | rs4659723 |
| MTR | rs4077829 |
| MTR | rs1805087 |
| MTR | rs4659743 |
