# Supplementary material for: Candidate gene analysis of spontaneous preterm delivery: New insights from re-analysis of a case-control study using case-parent triads and control-mother dyads
Source: BMC Med Genet. 2011 Dec 30;12:174. doi: 10.1186/1471-2350-12-174 (PMC3260094; doi:10.1186/1471-2350-12-174)
Supplement: Additional file 2 — Table S2 KEGG pathways examined. Table showing which KEGG pathways that have been examined in the study. [file 1471-2350-12-174-S2.DOC]

**Table S2.** **KEGG Pathways examined.**

| **KEGG pathway** | **Name of pathway** |
| --- | --- |
| hsa00010 | Glycolysis / Gluconeogenesis |
| hsa00020 | Citrate cycle (TCA cycle) |
| hsa00030 | Pentose phosphate pathway |
| hsa00031 | Inositol Metabolism |
| hsa00040 | Pentose and glucuronate interconversions |
| hsa00051 | Fructose and mannose metabolism |
| hsa00052 | Galactose metabolism |
| hsa00053 | Ascorbate and aldarate metabolism |
| hsa00061 | Fatty acid biosynthesis |
| hsa00062 | Fatty acid elongation in mitochondria |
| hsa00071 | Fatty acid metabolism |
| hsa00072 | Synthesis and degradation of ketone bodies |
| hsa00100 | Steroid biosynthesis |
| hsa00120 | Primary bile acid biosynthesis |
| hsa00130 | Ubiquinone and other terpenoid-quinone biosynthesis |
| hsa00140 | C21-Steroid hormone metabolism |
| hsa00150 | Androgen and estrogen metabolism |
| hsa00190 | Oxidative phosphorylation |
| hsa00220 | Urea cycle and metabolism of amino groups |
| hsa00230 | Purine metabolism |
| hsa00232 | Caffeine metabolism |
| hsa00240 | Pyrimidine metabolism |
| hsa00251 | Glutamate metabolism |
| hsa00252 | Alanine and aspartate metabolism |
| hsa00260 | Glycine, serine and threonine metabolism |
| hsa00271 | Methionine metabolism |
| hsa00272 | Cysteine metabolism |
| hsa00280 | Valine, leucine and isoleucine degradation |
| hsa00281 | Geraniol degradation |
| hsa00290 | Valine, leucine and isoleucine biosynthesis |
| hsa00300 | Lysine biosynthesis |
| hsa00310 | Lysine degradation |
| hsa00330 | Arginine and proline metabolism |
| hsa00340 | Histidine metabolism |
| hsa00350 | Tyrosine metabolism |
| hsa00360 | Phenylalanine metabolism |
| hsa00361 | gamma-Hexachlorocyclohexane degradation |
| hsa00363 | Bisphenol A degradation |
| hsa00364 | Fluorobenzoate degradation |
| hsa00380 | Tryptophan metabolism |
| hsa00400 | Phenylalanine, tyrosine and tryptophan biosynthesis |
| hsa00401 | Novobiocin biosynthesis |
| hsa00410 | beta-Alanine metabolism |
| hsa00430 | Taurine and hypotaurine metabolism |
| hsa00440 | Aminophosphonate metabolism |
| hsa00450 | Selenoamino acid metabolism |
| hsa00460 | Cyanoamino acid metabolism |
| hsa00471 | D-Glutamine and D-glutamate metabolism |
| hsa00472 | D-Arginine and D-ornithine metabolism |
| hsa00480 | Glutathione metabolism |
| hsa00500 | Starch and sucrose metabolism |
| hsa00510 | N-Glycan biosynthesis |
| hsa00511 | Other glycan degradation |
| hsa00512 | O-Glycan biosynthesis |
| hsa00520 | Amino sugar and nucleotide sugar metabolism |
| hsa00521 | Streptomycin biosynthesis |
| hsa00530 | Aminosugars metabolism |
| hsa00531 | Glycosaminoglycan degradation |
| hsa00532 | Chondroitin sulfate biosynthesis |
| hsa00533 | Keratan sulfate biosynthesis |
| hsa00534 | Heparan sulfate biosynthesis |
| hsa00550 | Peptioglycan biosynthesis |
| hsa00561 | Glycerolipid metabolism |
| hsa00562 | Inositol phosphate metabolism |
| hsa00563 | Glycosylphosphatidylinositol(GPI)-anchor biosynthesis |
| hsa00564 | Glycerophospholipid metabolism |
| hsa00565 | Ether lipid metabolism |
| hsa00590 | Arachidonic acid metabolism |
| hsa00591 | Linoleic acid metabolism |
| hsa00592 | alpha-Linolenic acid metabolism |
| hsa00600 | Sphingolipid metabolism |
| hsa00601 | Glycosphingolipid biosynthesis - lacto and neolacto series |
| hsa00602 | Glycosphingolipid biosynthesis - neo-lacto series |
| hsa00603 | Glycosphingolipid biosynthesis - globo series |
| hsa00604 | Glycosphingolipid biosynthesis - ganglio series |
| hsa00620 | Pyruvate metabolism |
| hsa00624 | 1- and 2-Methylnaphthalene degradation |
| hsa00625 | Tetrachloroethene degradation |
| hsa00627 | 1,4-Dichlorobenzene degradation |
| hsa00630 | Glyoxylate and dicarboxylate metabolism |
| hsa00632 | Benzoate degradation via CoA ligation |
| hsa00640 | Propanoate metabolism |
| hsa00641 | 3-Chloroacrylic acid degradation |
| hsa00643 | Styrene degradation |
| hsa00650 | Butanoate metabolism |
| hsa00670 | One carbon pool by folate |
| hsa00680 | Methane metabolism |
| hsa00710 | Carbon fixation in photosynthetic organisms |
| hsa00720 | Reductive carboxylate cycle (CO2 fixation) |
| hsa00730 | Thiamine metabolism |
| hsa00740 | Riboflavin metabolism |
| hsa00750 | Vitamin B6 metabolism |
| hsa00760 | Nicotinate and nicotinamide metabolism |
| hsa00770 | Pantothenate and CoA biosynthesis |
| hsa00780 | Biotin metabolism |
| hsa00785 | Lipoic acid metabolism |
| hsa00790 | Folate biosynthesis |
| hsa00791 | Atrazine degradation |
| hsa00830 | Retinol metabolism |
| hsa00860 | Porphyrin and chlorophyll metabolism |
| hsa00900 | Terpenoid backbone biosynthesis |
| hsa00902 | Monoterpenoid biosynthesis |
| hsa00903 | Limonene and pinene degradation |
| hsa00910 | Nitrogen metabolism |
| hsa00920 | Sulfur metabolism |
| hsa00930 | Caprolactam degradation |
| hsa00950 | Isoquinoline alkaloid biosynthesis |
| hsa00960 | Tropane, piperidine and pyridine alkaloid biosynthesis |
| hsa00970 | Aminoacyl-tRNA biosynthesis |
| hsa00980 | Metabolism of xenobiotics by cytochrome P450 |
| hsa00982 | Drug metabolism - cytochrome P450 |
| hsa00983 | Drug metabolism - other enzymes |
| hsa01030 | Glycan structures - biosynthesis 1 |
| hsa01031 | Glycan structures - biosynthesis 2 |
| hsa01032 | Glycan structures - degradation |
| hsa01040 | Biosynthesis of unsaturated fatty acids |
| hsa01061 | Biosynthesis of phenylpropanoids |
| hsa01430 | Cell Communication |
| hsa01510 | Neurodegenerative diseases |
| hsa02010 | ABC transporters |
| hsa03010 | Ribosome |
| hsa03020 | RNA polymerase |
| hsa03022 | Basal transcription factors |
| hsa03030 | DNA replication |
| hsa03050 | Proteasome |
| hsa03060 | Protein export |
| hsa03320 | PPAR signaling pathway |
| hsa03410 | Base excision repair |
| hsa03420 | Nucleotide excision repair |
| hsa03430 | Mismatch repair |
| hsa03440 | Homologous recombination |
| hsa03450 | Non-homologous end-joining |
| hsa04010 | MAPK signaling pathway |
| hsa04012 | ErbB signaling pathway |
| hsa04020 | Calcium signaling pathway |
| hsa04060 | Cytokine-cytokine receptor interaction |
| hsa04070 | Phosphatidylinositol signaling system |
| hsa04080 | Neuroactive ligand-receptor interaction |
| hsa04110 | Cell cycle |
| hsa04115 | p53 signaling pathway |
| hsa04120 | Ubiquitin mediated proteolysis |
| hsa04130 | SNARE interactions in vesicular transport |
| hsa04140 | Regulation of autophagy |
| hsa04150 | mTOR signaling pathway |
| hsa04210 | Apoptosis |
| hsa04310 | Wnt signaling pathway |
| hsa04330 | Notch signaling pathway |
| hsa04340 | Hedgehog signaling pathway |
| hsa04350 | TGF-beta signaling pathway |
| hsa04360 | Axon guidance |
| hsa04370 | VEGF signaling pathway |
| hsa04510 | Focal adhesion |
| hsa04512 | ECM-receptor interaction |
| hsa04514 | Cell adhesion molecules (CAMs) |
| hsa04520 | Adherens junction |
| hsa04530 | Tight junction |
| hsa04540 | Gap junction |
| hsa04610 | Complement and coagulation cascades |
| hsa04612 | Antigen processing and presentation |
| hsa04614 | Renin-angiotensin system |
| hsa04620 | Toll-like receptor signaling pathway |
| hsa04630 | Jak-STAT signaling pathway |
| hsa04640 | Hematopoietic cell lineage |
| hsa04650 | Natural killer cell mediated cytotoxicity |
| hsa04660 | T cell receptor signaling pathway |
| hsa04662 | B cell receptor signaling pathway |
| hsa04664 | Fc epsilon RI signaling pathway |
| hsa04670 | Leukocyte transendothelial migration |
| hsa04710 | Circadian rhythm - mammal |
| hsa04720 | Long-term potentiation |
| hsa04730 | Long-term depression |
| hsa04740 | Olfactory transduction |
| hsa04742 | Taste transduction |
| hsa04810 | Regulation of actin cytoskeleton |
| hsa04910 | Insulin signaling pathway |
| hsa04912 | GnRH signaling pathway |
| hsa04916 | Melanogenesis |
| hsa04920 | Adipocytokine signaling pathway |
| hsa04930 | Type II diabetes mellitus |
| hsa04940 | Type I diabetes mellitus |
| hsa04950 | Maturity onset diabetes of the young |
| hsa05010 | Alzheimer's disease |
| hsa05012 | Parkinson's disease |
| hsa05020 | Prion diseases |
| hsa05030 | Amyotrophic lateral sclerosis (ALS) |
| hsa05040 | Huntington's disease |
| hsa05050 | Dentatorubropallidoluysian atrophy (DRPLA) |
| hsa05060 | Prion disease |
| hsa05110 | Vibrio cholerae infection |
| hsa05120 | Epithelial cell signaling in Helicobacter pylori infection |
| hsa05130 | Pathogenic Escherichia coli infection - EHEC |
| hsa05131 | Shigellosis |
| hsa05210 | Colorectal cancer |
| hsa05211 | Renal cell carcinoma |
| hsa05212 | Pancreatic cancer |
| hsa05213 | Endometrial cancer |
| hsa05214 | Glioma |
| hsa05215 | Prostate cancer |
| hsa05216 | Thyroid cancer |
| hsa05217 | Basal cell carcinoma |
| hsa05218 | Melanoma |
| hsa05219 | Bladder cancer |
| hsa05220 | Chronic myeloid leukemia |
| hsa05221 | Acute myeloid leukemia |
| hsa05222 | Small cell lung cancer |
| hsa05223 | Non-small cell lung cancer |
| hsa05310 | Asthma |
| hsa05320 | Autoimmune thyroid disease |
| hsa05322 | Systemic lupus erythematosus |
| hsa05330 | Allograft rejection |
| hsa05332 | Graft-versus-host disease |
| hsa05340 | Primary immunodeficiency |
